# A Comprehensive Review of Functional Gel Polymer Electrolytes and Applications in Lithium-Ion Battery

**DOI:** 10.3390/gels10090563

**Published:** 2024-08-29

**Authors:** Md. Shahriar Ahmed, Mobinul Islam, Bikash Raut, Sua Yun, Hae Yong Kim, Kyung-Wan Nam

**Affiliations:** Department of Energy & Materials Engineering, Dongguk University, Seoul 04620, Republic of Korea; shahriar.che.sust@gmail.com (M.S.A.); knam@dongguk.edu (K.-W.N.)

**Keywords:** Li-ion battery, gel polymer electrolytes, fire safety, high nickel cathode, long cycling

## Abstract

The rapid expansion of flexible and wearable electronics has necessitated a focus on ensuring their safety and operational reliability. Gel polymer electrolytes (GPEs) have become preferred alternatives to traditional liquid electrolytes, offering enhanced safety features and adaptability to the design requirements of flexible lithium-ion batteries. This review provides a comprehensive and critical overview of recent advancements in GPE technology, highlighting significant improvements in its physicochemical properties, which contribute to superior long-term cycling stability and high-rate capacity compared with traditional organic liquid electrolytes. Special attention is given to the development of smart GPEs endowed with advanced functionalities such as self-protection, thermotolerance, and self-healing properties, which further enhance battery safety and reliability. This review also critically examines the application of GPEs in high-energy cathode materials, including lithium nickel cobalt manganese (NCM), lithium nickel cobalt aluminum (NCA), and thermally stable lithium iron phosphate (LiFePO_4_). Despite the advancements, several challenges in GPE development remain unresolved, such as improving ionic conductivity at low temperatures and ensuring mechanical integrity and interfacial compatibility. This review concludes by outlining future research directions and the remaining technical hurdles, providing valuable insights to guide ongoing and future efforts in the field of GPEs for lithium-ion batteries, with a particular emphasis on applications in high-energy and thermally stable cathodes.

## 1. Introduction

**Scheme 1:**
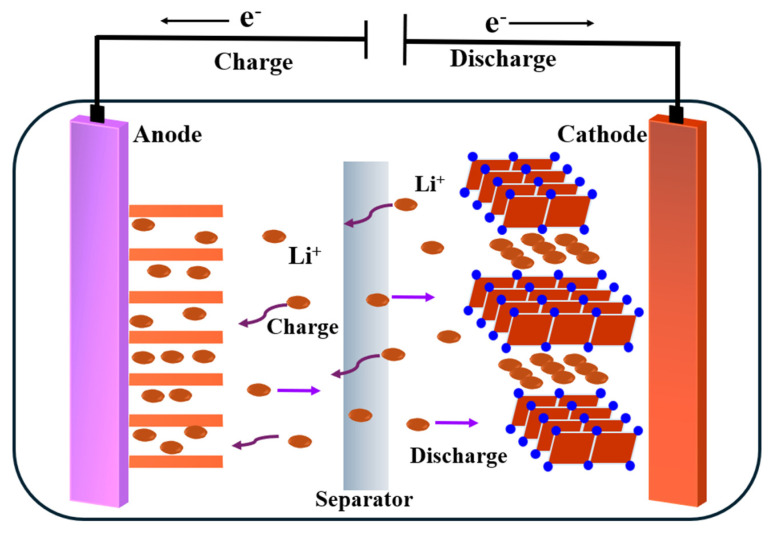
Schematic of conventional lithium-ion battery cathode design and lithium-ion transportation through the separator in charging and discharging process.

As environmental concerns from greenhouse gas emissions continue to rise and nonrenewable fossil fuel supplies wane, the adoption of renewable energy alternatives has become pivotal in scientific exploration and progress. Today, one of the significant hurdles that societies must overcome is the shift from the current energy framework, which is reliant on environmentally harmful and finite fossil fuels, to a new model centered on clean, enduring, and sustainable energy sources. Hence, an effective energy storage system is essential to ensuring a consistent energy supply. Among various energy storage solutions, electrochemical storage utilizing lithium-ion batteries stands out due to its high efficiency and extended cycle life [1]. Lithium-ion batteries (LIBs) represent a key advancement in electrochemical energy storage, utilizing lithium intercalating compounds. Over the past twenty years, they have become a major focus in research. Initially brought to the market by Sony Corporation in the 1990s, LIBs have since taken over the portable electronic device sector, powering laptops, mobile phones, and cameras, and recently electric buses and cars. Their desirable attributes—such as low weight, higher energy density, high open circuit voltage, rapid charging capabilities, low self-discharge rate, and environmental sustainability—make them an attractive option for electric vehicle technology [2,3,4,5,6]. Outlined in Scheme 1, the standard lithium-ion battery (LIB) comprises vital components, i.e., the cathode and anode, a nonaqueous electrolyte system, and a separator that averts direct electrode contact. The LIB’s functionality is contingent on lithium ions shifting between the electrodes. When charging, lithium ions traverse from cathode to anode via the electrolyte, facilitating current flow. Conversely, during discharge, the lithium ions reverse course, traveling from the anode back to the cathode.

Furthermore, an important safety consideration arises from the utilization of flammable organic solvents in LIBs, along with the issue of electrolyte leakage, which has hindered the widespread adoption of liquid electrolyte-based LIBs [7,8,9,10]. The presence of organic solvents introduces the potential risk of generating highly flammable gases such as carbon monoxide (CO), methane (CH_4_), ethylene (C_2_H_4_), ethane (C_2_H_6_), propylene (C_3_H_6_), and propane (C_3_H_8_). Addressing these safety concerns is crucial in enhancing the overall safety and reliability of lithium-ion battery technologies. When LIBs are exposed to overheating due to overcharging, the associated risks are heightened. Additionally, a significant concern revolves around the compatibility between electrodes and electrolytes, particularly linked to the utilization of high-voltage materials, predominantly found in cathodes, aimed at boosting the energy density of LIBs. This compatibility issue can lead to electrolyte degradation because of inadequate electrochemical stability at elevated voltage levels [11,12]. The harmony between electrodes and electrolytes depends on the electrochemical boundary of electrolytes. This means that ensuring seamless interactions between electrolytes and high-voltage cathode materials is a crucial aspect of electrolyte development, which requires comprehensive consideration. A compilation of incidents involving LIBs indicates that a majority of accidents stem from operational overheating, resulting in fires [13,14]. Consequently, there is a growing demand for the substitution of liquid organic electrolytes in LIBs to address safety concerns. Solid electrolytes, encompassing inorganic solid electrolytes and polymer electrolytes, have garnered significant interest as potential alternatives to liquid electrolytes. Furthermore, within the category of solid electrolytes, gel polymer electrolytes have emerged as a focal point for substituting liquid electrolytes, lauded for their inherent characteristics like security, flexibility, and dependability [12]. Table 1 represents the applications of gel polymer electrolytes in LIBs based on the polymer backbone. Polymer electrolytes can be broadly categorized into two types: solid polymer electrolytes (SPEs) that do not contain any solvent, and gel polymer electrolytes (GPEs). The line between SPEs and GPEs can sometimes be blurred, as incorporating plasticizers into SPEs can make them exhibit characteristics similar to GPEs. In this review, we define safety, particularly fire safety, through both quantitative and qualitative measures. Quantitatively, we evaluate safety using criteria like ignition temperature, flammability indices, and heat release rates, comparing these values against recognized safety standards. Qualitatively, we assess materials based on their behavior under extreme conditions, ability to self-extinguish, and performance in real-world fire hazard scenarios. This comprehensive approach ensures informed decisions regarding material suitability for various applications. We are also considering incorporating references to specific safety standards to strengthen our findings.

### 1.1. Solid Polymer Electrolytes

**Scheme 2:**
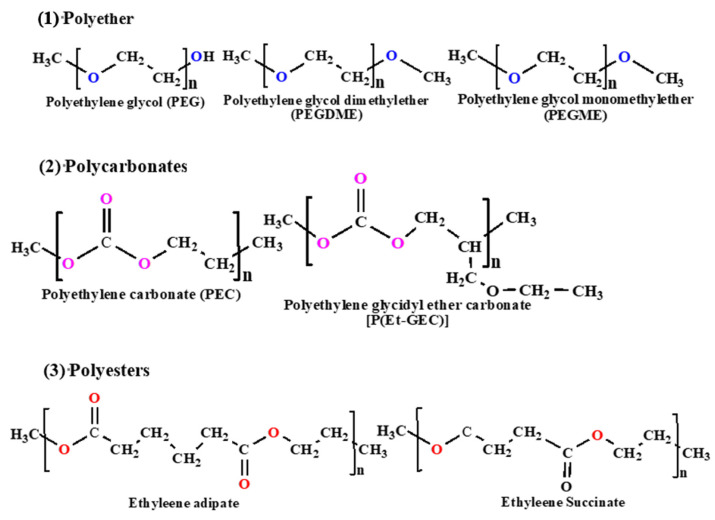
Common polymer backbone for solid polymer electrolytes.

**Scheme 3:**
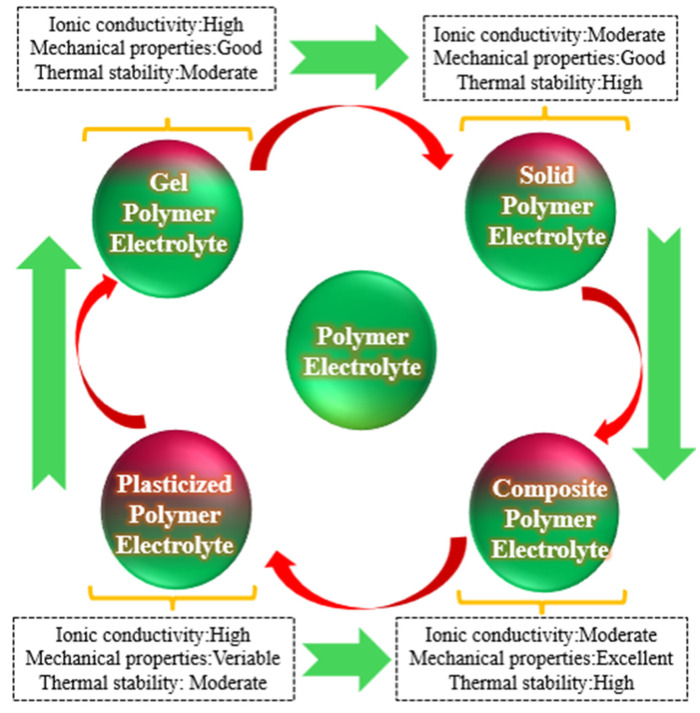
Comparison of gel polymer electrolytes and other in terms of ionic conductivity and mechanical properties, polymer electrolytes properties, and thermal stability.

In the realm of solid-state battery technology, the formulation of solid-polymer electrolytes (SPEs) represents a crucial advancement achieved by embedding lithium salts within a polymeric matrix to bolster ionic conductivity. Within the intricate structure of SPEs, the process of lithium salt dissolution unfolds through a sophisticated interplay between the Li ion and the electron-rich polymer backbone [20], a dynamic interaction that plays a pivotal role in fostering enhanced ionic conductivity within the system, as evidenced by prior research findings [21,22]. The transition from conventional liquid electrolytes to SPEs in LIBs heralds a paradigm shift, offering a myriad of benefits ranging from nonvolatility to heightened mechanical flexibility and elevated safety standards, underscoring the transformative potential of solid-state battery technologies [20,23]. Delving deeper into the spectrum of polymer backbones utilized in SPE design for LIB applications reveals a rich landscape of materials, among which polyethylene oxide (PEO) emerges as a standout candidate. PEO’s distinctive composition, characterized by flexible segments of ethylene oxide and atoms of ether oxygen endowed with potent donor characteristics, renders it an ideal solid solvent for lithium salts, enabling seamless solvation of Li^+^ cations essential for battery operation and efficiency. The common polymer backbone for solid polymer electrolytes for LIBs is represented in Scheme 2. Despite its inherent virtues, the solid nature of polymer frameworks presents a unique challenge, impeding SPEs from establishing a seamless interface with electrodes akin to that observed in liquid electrolytes. Consequently, the interfacial interaction between SPEs and electrodes is confined to the discrete contact area on the electrode surface, giving rise to heightened interfacial impedance that can directly influence the battery’s overall efficiency and lifespan system. Gel polymer electrolytes and other polymer electrolytes’ ionic conductivity, mechanical properties, and thermal stability comparison are shown in Scheme 3.

### 1.2. Gel Polymer Electrolytes

**Scheme 4:**
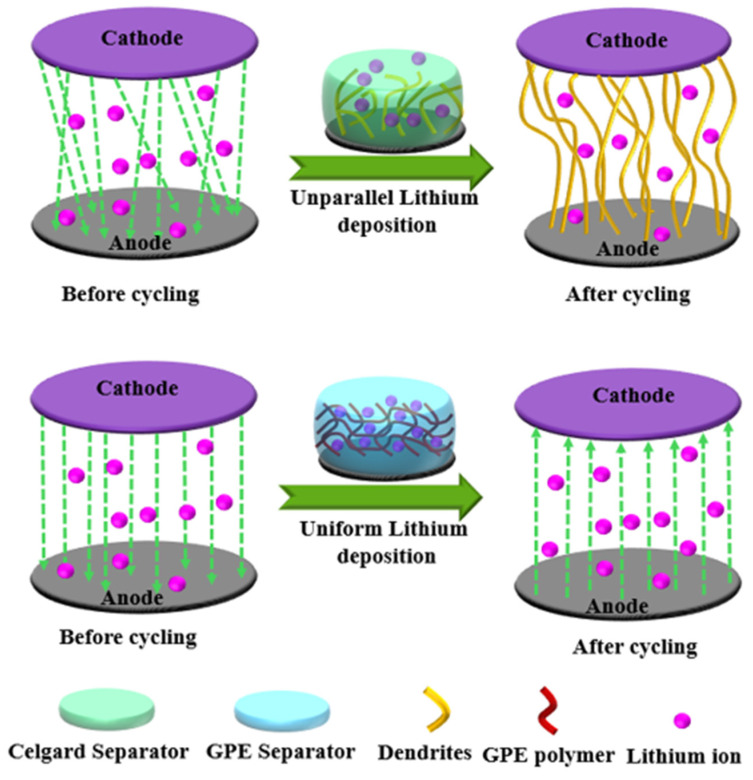
Gel polymer electrolyte application in lithium-ion battery to suppress dendrite formation from lithium metal anode to cathode.

Gel polymer electrolytes have garnered considerable interest in the realm of energy storage capabilities because of their distinctive characteristics and wide-ranging potential in diverse electrochemical devices, as these electrolytes comprise a polymer framework infused when combined with a liquid electrolyte, resulting in the creation of a gel-like structure. This gel matrix plays a pivotal role in enabling efficient ion transport, which facilitates effective charge transfer in batteries, fuel cells, and supercapacitors. In contrast with solid-polymer electrolytes (SPEs), gel polymer electrolytes (GPEs) exhibit enhanced promise for practical implementation in lithium-ion batteries (LIBs) by combining high ionic conductivity from liquid electrolytes with favorable mechanical properties akin to SPEs [24]. Feuillade and Perche introduced the idea of gel-based polymer electrolytes back in 1975. They were pioneers in showcasing the application of GPEs, specifically using coke/GPE/LiMn_2_O_4_ in LIBs. The GPE formulation involved a poly(vinylidene fluoride)-co-hexafluoropropylene (PVDF-HFP) copolymer matrix, incorporating a gel formed by a LiPF_6_ solution in ethylene carbonate (EC)/dimethyl carbonate (DMC) [25]. Gel-based polymer electrolytes are typically classified into two main categories: physical gels and chemical gels. Physical gels are favored for their straightforward preparation process, flexibility, and robust mechanical stability. In physical gels, the liquid electrolytes are contained within a polymer matrix without any chemical bonding taking place between the polymer and the solvent. Conversely, chemical gels involve the incorporation of a crosslinker, facilitating the creation of chemical bonds between the functional groups of the polymer and the crosslinking agent. Chemical gels, renowned for their enduring gel structure and superior mechanical resilience when contrasted with physical gels, present elevated stability and can be customized for specific attributes by adjusting variables like chemical composition and the density of crosslinking. Both physical and chemical gels offer a range of advantages for polymer electrolytes, facilitating high ionic conductivity by enveloping a liquid electrolyte within the gel matrix. This containment of the liquid electrolyte encapsulated within the gel framework reduces the likelihood of leaks, ultimately enhancing the safety of electrochemical devices. The amalgamation of ionic conductivity, mechanical strength, and safety features endorses polymer electrolytes with gel-like properties, which present a promising contender for advanced electrochemical devices, affording heightened performance and endurance, which in turn contributes to the enhancement of efficiency and safety standards in LIBs. In the realm of LIBs, gel-based polymer electrolytes, composed of a polymer matrix saturated with a liquid electrolyte, serve a pivotal role in facilitating the movement of lithium ions between the cathode and the anode. A fundamental function of these electrolytes is to furnish a stable and conductive environment for ion transport within LIBs. The gel structure of these electrolytes is adept at accommodating the volume fluctuations that transpire during the lithium-ion insertion and extraction phases. This adaptability helps alleviate mechanical strain on the electrode materials, thereby diminishing the likelihood of electrode deterioration and augmenting the overall longevity of the battery system. Furthermore, gel-based polymer electrolytes play a critical role in enhancing the safety profile of LIBs. By impeding the formation of lithium dendrites, which have the potential to trigger short circuits and jeopardize battery integrity, the gel matrix contributes significantly to maintaining the safety and reliability of lithium-ion batteries [26]. Scheme 4 represents the application of gel polymer electrolyte (GPE) in LIBs to suppress dendrite formation from lithium metal anode to cathode. Multiple polymer matrices, like PEO and PAN, PVDF, PMMA, PVA, PVC, and various others, have been the subject of in-depth research to determine their suitability for implementation in GPEs within the domain of LIBs [20,25]. Although GPEs exhibit superior performance regarding ionic conductivity compared with SPEs, they introduce additional complexities due to the interplay between liquid and solid electrolyte components [1,10]. This review delves into the essential elements of GPEs, covering their core requirements, notable properties, long-lasting cycling stability, and crucial thermal safety features, particularly in the context of charging and utilization in high-nickel cathode batteries aimed at advancing lithium-ion battery technology. Subsequently, this article systematically explores the recent advancements in GPE research for LIBs, focusing on how the structural design of the polymer matrix contributes to enhanced electrochemical performance. Finally, this discussion extends to other potential polymer alternatives and outlines the envisioned future trajectory of GPEs within LIB applications [27].

## 2. The Creation of the Polymer Framework in Gel Polymer Electrolytes (GPEs)

In the fabrication of GPEs, a standard procedure typically involves two primary phases, the development of porous polymer membrane structures, which are then activated using a liquid organic electrolyte. This systematic approach interlaces the performance of GPEs intricately with the distinctive attributes of the chosen polymer host. Various conventional polar polymers, such as a polymer widely recognized as polyethylene oxide (PEO), a subset of fluoropolymer commonly referred to by the acronym PVDF, a copolymer blend of vinylidene fluoride and hexafluoropropylene known as PVDF-HFP, a polymer often identified by the abbreviation PMMA, and an acrylic polymer termed PAN, have been frequently utilized as the foundational component for GPE matrices. This section delves into offering a comprehensive overview of the recent strides made in leveraging these well-established polymer structures for GPE applications. Moreover, it delves deeper into the exploration of innovative eco-conscious and biodegradable polymer frameworks, aligning closely with the prevailing emphasis on sustainability and the ongoing efforts to foster environmentally friendly material development within the sphere of polymer electrolytes [28].

## 3. Polymer Backbone-Based GEPs in Lithium-Ion Battery

### 3.1. PVDF-Based GPEs

The development of GPEs for lithium-ion batteries has been significantly propelled by focused research utilizing polyvinylidene fluoride (PVDF) and its copolymers, which are celebrated for their exceptional mechanical, chemical, and electrochemical properties. PVDF serves as an excellent matrix for GPEs due to its high dielectric constant, flexibility, and stability under electrochemical conditions, making it adaptable for a variety of ionic conductors. Researchers aim to optimize lithium-ion transport by manipulating the crystalline and amorphous phases of PVDF, improving ion transference numbers through strategic intermolecular interactions. These advancements not only address challenges like thermal stability and mechanical integrity but also pave the way for scalable and efficient energy storage solutions. Through these studies, PVDF-based GPEs demonstrate potential to enhance battery capacity, longevity, and efficiency while adhering to essential safety and environmental standards, marking a significant step forward in energy technology innovations.

**Figure 1:**
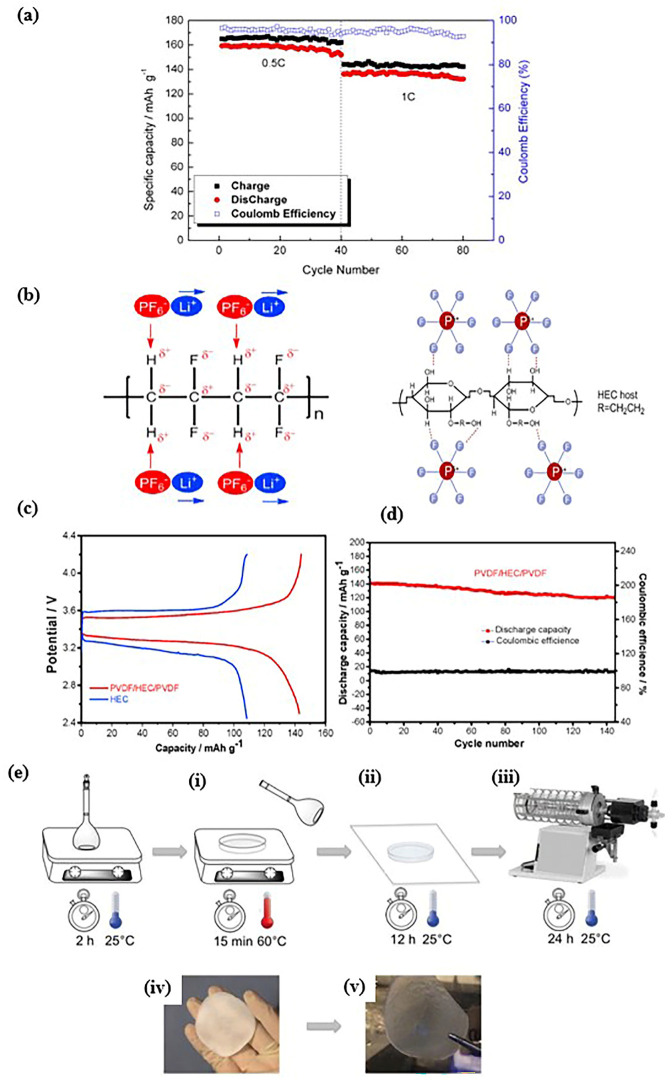
(**a**) The charging/discharging/discharge cycle functionality of the Li/ILGPE/LiFePO_4_ cell at 0.5 C and 1 C rates at 20 °C, reproduced with permission from ref. [29], Elsevier. (**b**) Interactions occurring in the GPEs: (i) between PVDF and LiPF_6_ and (ii) between LiPF_6_ and the hydroxyl groups in the HEC host. LiFePO_4_ positive electrode performance with HEC and the composite membrane made of PVDF/HEC/PVDF using 1 mol L^−1^ LiPF_6_ electrolyte, Li metal counter electrode: (**c**) charge–discharge at 0.2 C, (**d**) cycling behavior at 0.2 C; reproduced with permission from ref. [30], Elsevier. (**e**) Outlines of GPE membrane preparation: (i) dissolve PVDF in EC: DMC (20:80), stirring 2 h, (ii) heat, pour into Petri disc, (iii) age overnight, (iv) vacuum dry 3 h, (v) display dry PVDF precursor, reproduced with permission from ref. [31], Elsevier.

P. Yang and team conducted a study on a gel polymer electrolyte (GPE) using polyvinylidene fluoride-co-hexafluoropropylene ((PVDF-HFP)) and 1-butyl-4-methylpyridinium bis(trifluoromethanesulfonyl)imide (B_4_MePyTFSI) for lithium-ion battery applications. By employing solution casting, they developed GPEs showing enhanced ionic conductivity. Additionally, the electrochemical stability range of the ionic liquid gel polymer electrolyte (ILGPE) was around 5.5 V versus Li^+^/Li at 20 °C. When employed in lithium batteries, this ILGPE demonstrated a capacity of about 160 mAh g^−1^, displaying robust cycle reliability and suitability with LiFePO_4_ electrodes. Minimal changes in interfacial resistance after 10 cycles suggested excellent electrode matching. These findings suggest the possibility of ILGPEs for enhancing rechargeable lithium-air batteries through improved conductivity, stability, and overall performance, as shown in Figure 1a [29]. 

M.Y. Zhang et al. developed a sandwich-structured gel polymer electrolyte (GPE) utilizing polyvinylidene fluoride (PVDF) and hydroxyethyl cellulose (HEC) for lithium-ion batteries [30]. The PVDF/HEC/PVDF membrane was crafted via an efficient electro-spinning technique, demonstrating a constant discharge capacity of 140 mAh g^−1^ over 140 cycles in Li//LiFePO_4_ cells using this GPE. Additionally, the non-combustible properties of PVDF and HEC enhance safety, positioning this GPE as a reliable and scalable option for lithium-ion battery production. The polarity of the PVDF structure improves the Li^+^ ion transference number through the formation of robust H-F hydrogen bonds with PF_6_^−^ anions, impeding their mobility. Similarly, within the HEC host, hydrogen bonding interactions between PF_6_^−^ anions and ^−^OH groups restrict anion movement, collectively enhancing the Li^+^ ion mobility number (as shown in Figure 1b). LiFePO_4_ positive electrode charge–discharge at 0.2 C using HEC and PVDF/HEC/PVDF GPE. LiFePO_4_ capacity with PVDF/HEC/PVDF GPE is 140 mAh g^−1^, surpassing HEC GPE (110 mAh g^−1^), with slight capacity reduction after 60 cycles. Cycling and charge efficiencies of PVDF/HEC/PVDF GPE remain stable after 145 cycles, sustaining nearly 100% efficiency (as shown in Figure 1c,d).

C. Fasciani et al. have introduced an advanced gel polymer electrolyte (GPE) based on poly(vinylidene difluoride) (PVDF) optimized for LIBs [31]. This GPE, crafted as a dry and pliant film under standard conditions, ensures structural integrity by incorporating crystallized EC-based solvent to prevent collapse. With a design conducive to easy handling and scalable production, it employs a micrometric polymer network as the foundational gel matrix. Electrochemical assessments have validated stable lithium electrode–gel electrolyte interaction and commendable lithium transference properties. Through cyclic testing involving lithium iron phosphate (LiFePO_4_) and graphite electrodes, this innovative GPE has demonstrated efficiency as a cost-effective and environmentally friendly energy storage solution for LIBs. The GPE membrane was fabricated according to the protocol outlined in Figure 1e.

**Figure 2:**
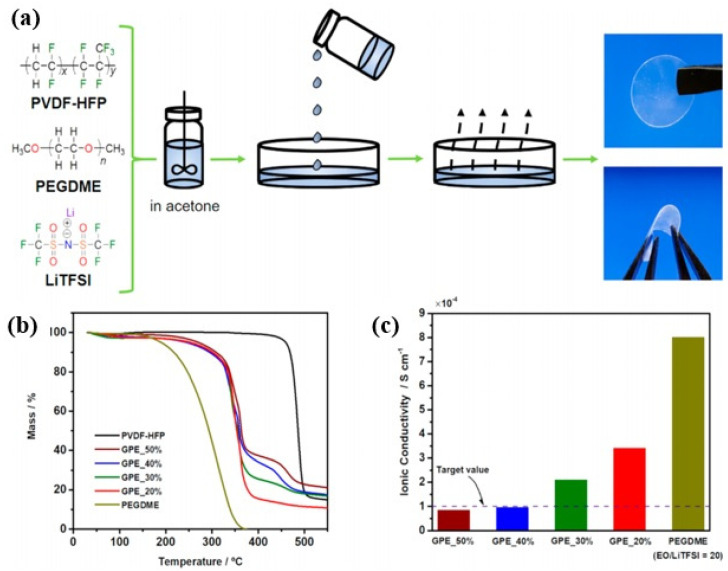
(**a**) Schematic illustration of preparation process, (**b**) TGA results, and (**c**) ionic conductivity at RT of GPEs with different percentages of PVDF-HFP content; adopted from ref. [33].

A cutting-edge composite membrane has been devised by combining electrospun poly(vinylidene fluoride) (PVDF) and lithium polyvinyl alcohol oxalate borate (LiPVAOB) materials, showcasing remarkable safety attributes and robust mechanical properties [32]. This innovative membrane boasts a lithium-ion transport number of 0.58 at ambient temperature, which is double the value found in commercial separators at 0.27. The SEM analysis reveals a distinct rough surface and compact inner structure of the LiPVAOB membrane, leading to minimal electrolyte absorption. When paired with electrospun PVDF fibers, a porous PVDF layer is formed with uniform pore sizes under 2 μm. The membrane exhibits a well-defined trilayer structure approximately 70 μm thick. Flammability assessments demonstrate superior flame-retardant qualities of the PVDF-LiPVAOB composite compared to the flammable Celgard 2730 separator. Julen Castillo devised a straightforward and scalable method to create a gel polymer electrolyte (GPE) incorporating the safe plasticizer polyethylene glycol dimethyl ether (PEGDME) combined with a poly (vinylidene fluoride-co-hexafluoropropylene) (PVDF-HFP) matrix. Schematic illustration of the preparation process (as shown in Figure 2a), TGA results (Figure 2b), and ionic conductivity at RT of GPEs with different percentages of PVDF-HFP content (Figure 2c) [33].

**Figure 3:**
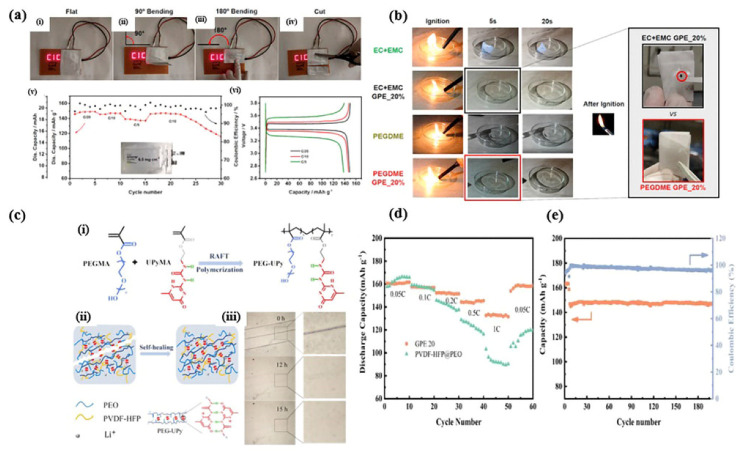
(**a**) Flexibility and safety tests of the Li^0^/GPE_20%/LFP pouch cell using LED lighting: (i) initial state, (ii) at 90°, (iii) at 180°, and (iv) post-cutting. It also includes (v) battery performance and (vi) charge/discharge characteristics at different C-rates. (**b**) Illustrates the flammability test conducted on EC + EMC and PEGDME solvents alongside their respective gel polymer electrolytes; adopted from ref. [33]. (**c**) (i) Represents the schematic synthesis process of the brush-like copolymer PEG-UPy. (ii) Illustrates the formation of PP-PU along with its self-healing mechanism at the surface of the cut. (iii) Presents optical images illustrating the self-healing process of PP-20PU samples under normal conditions. (**d**) Compares the discharge-specific capacity of Li/GPE-20/LFP and Li/PVDF-HFP@PEO/LFP configurations cells across different rates. (**e**) Demonstrates the cycling efficiency and coulombic efficiency of Li/GPE-20/LFP cells when tested at 1 C. Reprinted with permission from [34]. Copyright © 2022, American Chemical Society.

The GPE membranes were produced through a one-pot solvent-casting process. This GPE exhibits exceptional safety properties, including nonflammability and remarkable thermal stability of up to 250 °C, maintaining a retention rate of 98% after 60 cycles in a coin cell setup. Furthermore, a prototype pouch cell achieving approximately 19 mAh g^−1^ at a C/10 rate demonstrates outstanding security, adaptability, and resilience to bending and cutting. The flexibility, safety, and possibilities of Li^0^/GPE_20%/LFP pouch cells were showcased by powering LED lamps (as shown in Figure 3a). In the flammability test, GPE_20% outperformed GPE with (EC + EMC), which ignited instantly and burned for over 20 s. GPE with (EC + EMC) did not sustain combustion, showing minimal burning. This underscores the effectiveness of the polymer matrix in confining liquid electrolytes and highlights safety benefits (as shown in Figure 3b).

Researchers have developed an innovative gel polymer electrolyte (GPE) created by blending a PEG-UPy copolymer into a polymer matrix made of PVDF-HFP and PEO. This GPE features a unique quadruple hydrogen bonding network formed by UPy units, which grants it self-healing properties at 25 °C [34]. These self-healing capabilities are vital for preventing damage from lithium dendrite growth, thus enhancing the safety and longevity of lithium-ion batteries (as shown in Figure 3c). Batteries with GPE-20 exhibited a specific capacity of 163 mAh g^−1^ at a 1 C rate and maintained a charge efficiency of 97.3% over 200 cycles (as shown in Figure 3d,e). This advancement in polymer electrolyte technology promises improved efficiency and safety for lithium-ion batteries. Additionally, the team synthesized PEGMA/UPyMA copolymers with different UPy unit concentrations using the RAFT polymerization method. P. Zhang et al. developed a PVDF-HFP-PEO-SiO_2_ composite membrane with a porous structure utilizing the immersion precipitation technique [35]. The finger-like pores and elevated porosity of the membrane significantly improved liquid electrolyte uptake, resulting in enhanced ionic conductivity and lithium-ion transference. This membrane demonstrated impressive cycling performance, achieving a capacity of 147.7 mAh g^−1^ after 200 cycles with a LiFePO_4_ cathode. It effectively inhibited lithium dendrite growth and maintained excellent compatibility with lithium metal. With its simple fabrication process, environmental benefits, and low-cost production, this innovative GPE shows significant potential for efficient and secure lithium-ion batteries.

### 3.2. Cellulose Gel Membrane Gel Polymer Electrolyte

**Figure 4:**
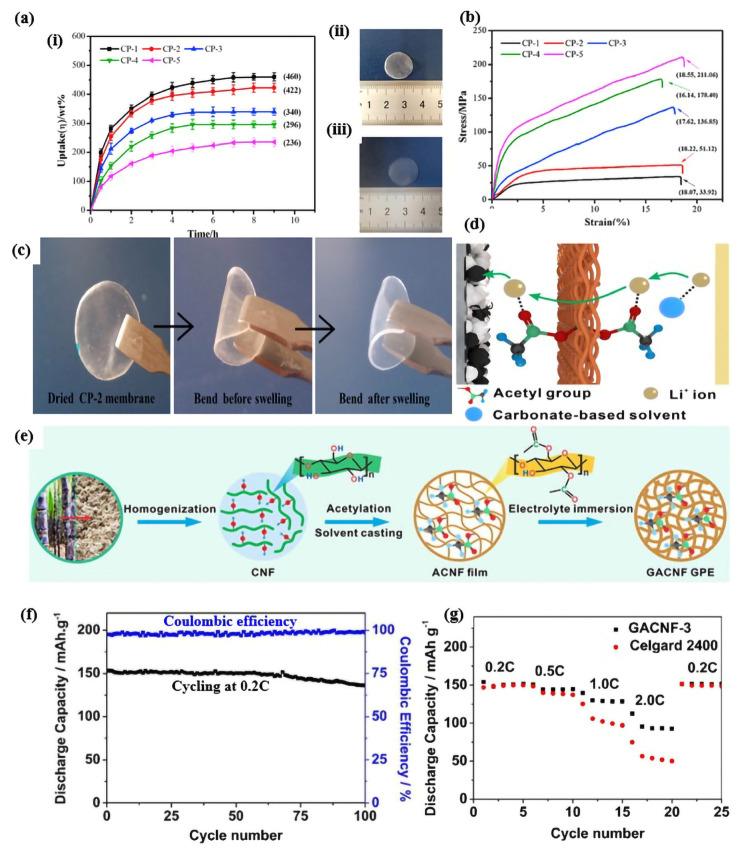
(**a**) (i) Shows the absorption of liquid electrolyte by composite membranes; optical photographs of the CP-2 membrane (ii) prior to and (iii) following immersion in the liquid electrolyte. (**b**) Illustrates the stress–strain characteristics of the composite membranes. (**c**) Illustrates the bending test results of the CP-2 membrane both before and after it has swelled; reproduced with permission from ref. [37], Elsevier. (**d**) Offers a schematic diagram of lithium-ion movement within the GACNF GPE. (**e**) Details the preparation process for both the ACNF film and the GACNF GPE. (**f**) Presents the performance data for the Li/GACNF-3 GPE/LiFePO_4_ cell at rates of 0.2, 0.5, 1.0, and 2.0 C. (**g**) Summarizes the cycling performance of the Li/GACNF-3 GPE/LiFePO4 cell at a rate of 0.2 C; reproduced with permission from ref. [38], Elsevier.

Cellulose-based GPEs represent an innovative approach to enhancing the performance of lithium-ion batteries. As a naturally abundant and renewable polymer, cellulose offers several advantages for use in GPEs, including sustainability, biodegradability, and excellent mechanical properties. When used as a matrix for gel electrolytes, cellulose can form a robust network capable of retaining liquid electrolytes, thus ensuring high ionic conductivity and mechanical integrity. The incorporation of cellulose in GPEs aims to overcome typical challenges associated with traditional polymer electrolytes, such as limited thermal and chemical stability. By introducing functional groups through modifications, cellulose can enhance the interaction between the electrolyte components and facilitate efficient ion transport. For instance, the presence of hydroxyl groups in cellulose allows for hydrogen bonding with ionic species, improving ion mobility and transference numbers. Through techniques like electrospinning or solution casting, cellulose can be processed into thin flexible membranes that are suitable for integration with various electrode materials. These membranes offer not only high ionic conductivity but also adherence to safety and environmental standards due to their non-toxic and non-flammable nature. A cellulose gel membrane that is both mechanically strong and environmentally friendly has been created through a straightforward solution casting technique followed by a one-step crosslinking process. Incorporating a 5% crosslinker, the cellulose membrane showcases a strong tensile strength at fracture, measuring 14.61 MPa, and outstanding electrochemical performance, featuring a significant electrolyte uptake of 540%, compatibility with lithium electrodes, and excellent electrochemical stability. The constructed cell exhibits a discharge capacity of 145 mAh g^−1^ after the first cycle at a 0.2 C rate and retains 90% capacity after 50 cycles. This natural polymer membrane is poised to serve as a high-safety, affordable, and environmentally friendly gel polymer electrolyte for lithium-ion batteries [36]. Figure 4a shows the absorption of the liquid electrolyte values and optical images of the membranes.

L. Zhao and their team investigated a flexible and durable cellulose/PEG-based gel polymer electrolyte (GPE) formulated for lithium-ion batteries. This GPE was developed using a one-step crosslinking method that integrates polyethylene glycol (PEG) into a crosslinked cellulose structure [37]. The material exhibited exceptional tensile strength, ranging from 33.92 MPa to 211.06 MPa, and swelling behavior (as shown in Figure 4b,c), and displayed notable bending resistance with PEG content between 2.5% and 20%. At a 5% PEG concentration, the assembled Li/GPE/NCM523 batteries achieved an initial discharge capacity of 159.3 mAh g^−1^ and a coulombic efficiency of 85.52% at 0.2 C. The GPE also demonstrated excellent compatibility with electrodes, suggesting its potential for cost-effective and biodegradable use in lithium-ion batteries. Efficient ion transport and liquid electrolyte retention are crucial for achieving high ionic conductivity in GPEs. 

W. Wang and his team developed an innovative nanocellulose-based gel polymer electrolyte (GPE) customized for lithium-ion batteries. By acetylating cellulose nanofibers (CNFs) and using solvent casting, followed by electrolyte immersion, they created GPE films with high electrolyte uptake (301%) and noteworthy mechanical strength, flexibility, and thermal stability. The movement of lithium ions within the GACNF GPE is shown in Figure 4d. The preparation of ACNF films involved solvent casting followed by immersion in liquid electrolyte, resulting in variants labeled ACNF-1, ACNF-2, and ACNF-3. Figure 4e provides a diagram of the preparation process and subsequent analysis of battery properties. Test results showed that the Li/GPE/LiFePO_4_ battery retained 88.8% capacity following 100 cycles at a 0.2 C rate. Additionally, the GACNF-3 demonstrated a rate capacity on par using the Celgard 2400 separator, as depicted in Figure 4f,g. These findings underscore the potential of acetylated CNF-based GPEs in high-efficiency lithium-ion batteries, pointing towards advances in quasi-solid and solid polymer electrolytes [38].

Xiao and his team investigated using methyl cellulose (MC) as a gel polymer electrolyte used in lithium-ion batteries, highlighting its robust properties and stability for enhanced safety. The MC gel membrane showed slightly higher ionic conductivity than the Celgard 2730 separator combined with liquid electrolyte. Tests with LiFePO_4_ and Li metal revealed the MC membrane’s superior performance [39]. The MC membrane demonstrated a specific capacity of around 130 mAh g^−1^ at 0.2 C, slightly exceeding the commercial separator’s capacity of about 126 mAh g^−1^. Cycling tests using the gel polymer electrolyte at 0.2 C exhibited excellent performance, comparable to the commercial separator. Notably, even after 40 cycles at 0.2 C, there was no significant capacity degradation.

### 3.3. Methacrylamide Based GPEs

A novel gel polymer electrolyte was developed, consisting of a poly(methacrylamide) derivative with a tri(cyanoethoxymethyl) group (PMCA), poly(ethylene glycol) dimethacrylate (PGM400), lithium bis(trifluoromethylsulfonyl)amide (LiTFSA), and propylene carbonate (PC). This electrolyte was synthesized using photo-initiated radical polymerization. The gel polymer electrolytes exhibited excellent oxidation stability, reaching about 4.6 V against a lithium electrode, facilitating reversible lithium plating and stripping on a nickel electrode. Additionally, the PMCA-GM400-based gel polymer electrolytes were transparent, flexible, and had glass transition temperatures (Tg) ranging from 192 K to 202 K, making them promising options for application in lithium secondary batteries [40]. M.M. Rao and collaborators conducted a study on PE-supported P(AN-co-MMA) gel polymer electrolytes for lithium-ion batteries. They synthesized a copolymer, poly (acrylonitrile-co-methyl methacrylate) (P(AN-co-MMA)), by varying the AN to MMA ratios through solution polymerization. The resulting gel polymer electrolyte, with PE-supported copolymer at a 4:1 AN to MMA ratio, exhibited impressive ionic conductivity at room temperature and stability up to 270 °C. This electrolyte demonstrated good compatibility with lithium-ion battery electrodes and showed promising characteristics for battery performance and cyclic stability. The research underscored the importance of the AN to MMA ratio in determining the properties of the electrolyte [41]. P. Isken et al. devised a methacrylate-based gel polymer electrolyte through free radical polymerization, combining oligo (ethylene glycol) methyl ether methacrylate (OEGMA) and benzyl methacrylate (BnMA) [42]. OEGMA’s ethylene glycol side chain facilitated interaction with the liquid electrolyte, improving its retention in the GPE, while BnMA enhanced mechanical stability. The polymer displayed exceptional liquid electrolyte retention, absorbing up to 400% of its weight, and maintained adequate mechanical integrity to function as a separator in lithium-ion batteries.

The selection of a polymer system containing OEGMA and CCMA for high-performance gel polymer electrolytes was based on its effective interaction and retention of liquid electrolytes. This system combines flexibility from OEGMA and mechanical stability from CCMA. The polymer exhibits exceptional thermal stability and ease of handling, leading to a gel electrolyte with enhanced conductivity and reliable cycling performance in full cells paired with NCM/graphite. Recognized as a promising electrolyte for lithium-ion battery applications, it integrates robust electrochemical properties with enhanced safety features [43].

**Figure 5:**
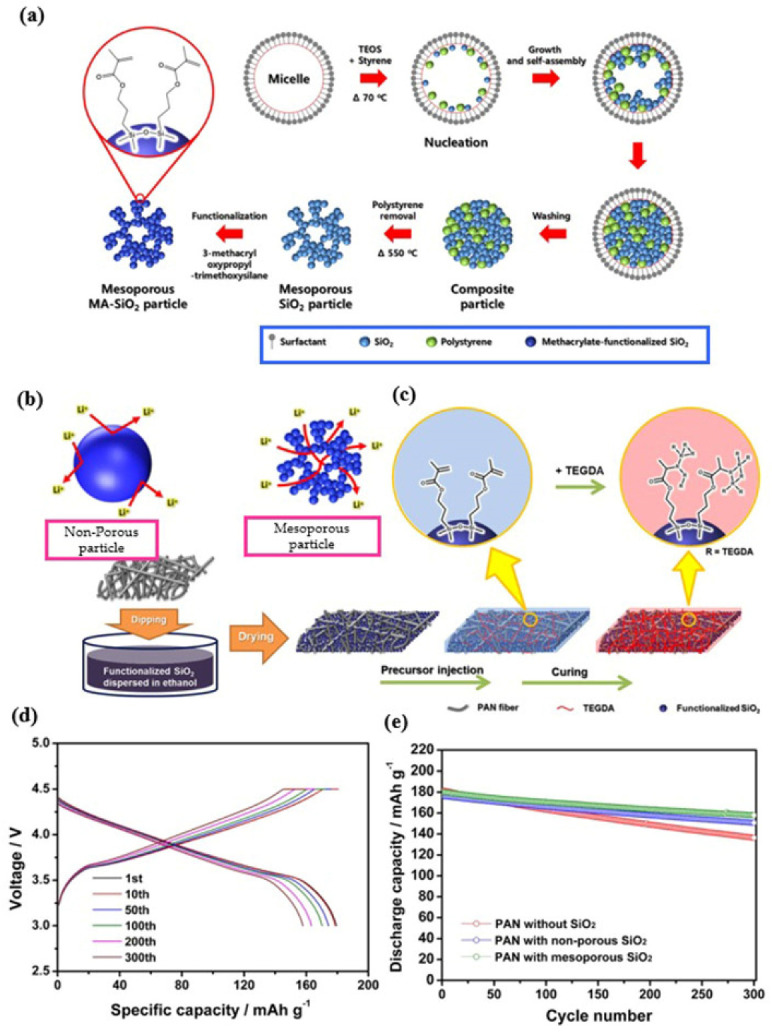
(**a**) Synthesis of mesoporous MA-SiO_2_ particles. (**b**) Varied lithium-ion transport characteristics with non-porous and mesoporous MA-SiO_2_ particles in the composite gel polymer electrolyte. (**c**) Development of the crosslinked composite gel polymer electrolyte utilizing a fibrous PAN membrane, mesoporous MA-SiO_2_ nanoparticles, and a gel electrolyte precursor featuring TEGDA. (**d**) Charge and discharge profiles of a lithium-ion polymer cell utilizing a crosslinked composite gel polymer electrolyte with mesoporous MA-SiO_2_ nanoparticles. (**e**) Discharge capacities of lithium-ion polymer cells using various electrolytes at 25 °C, adopted from ref. [44].

W.-K. Shin et al. created a crosslinked composite gel polymer electrolyte to boost the safety and reliability of lithium-ion polymer cells. Mesoporous SiO_2_ nanoparticles with reactive methacrylate groups were synthesized and incorporated within a fibrous polyacrylonitrile membrane [44]. Acting as crosslinking sites, these nanoparticles directly interacted with gel electrolyte precursors that included tri(ethylene glycol) diacrylate, resulting in a high ionic conductivity crosslinked composite gel polymer electrolyte incorporating favorable interfacial properties. Furthermore, the mesoporous SiO_2_ particles acted as HF scavengers, reducing HF content in the electrolyte at high temperatures and leading to significantly improved cycling performance in lithium-ion polymer cells when utilizing the crosslinked composite gel polymer electrolytes containing methacrylate-functionalized mesoporous SiO_2_ nanoparticles, particularly effective at high temperatures. Figure 5a demonstrates the synthesis scheme for mesoporous MA-SiO_2_ nanoparticles. Figure 5b,c showcase the different lithium-ion transport behaviors with non-porous and mesoporous MA-SiO_2_ particles within the composite gel polymer electrolyte and the synthesis of the crosslinked composite gel polymer electrolyte using a fibrous PAN membrane, mesoporous MA-SiO_2_ nanoparticles, and a TEGDA-containing gel electrolyte precursor. Figure 5d shows voltage profiles of a lithium-ion polymer cell incorporating a crosslinked composite gel polymer electrolyte embedded with mesoporous MA-SiO_2_ particles. Initially, the cell delivered a discharge capacity of 179.5 mAh g^−1^, calculated based on the active material LiNi_1/3_Co_1/3_Mn_1/3_O_2_. After 300 cycles, the discharge capacity was reduced to 157.9 mAh g^−1^, representing 88.0% of the initial capacity (Figure 5e). 

### 3.4. Fluorinated Gel Polymer Electrolyte Derived from PEGDA Facilitated

**Figure 6:**
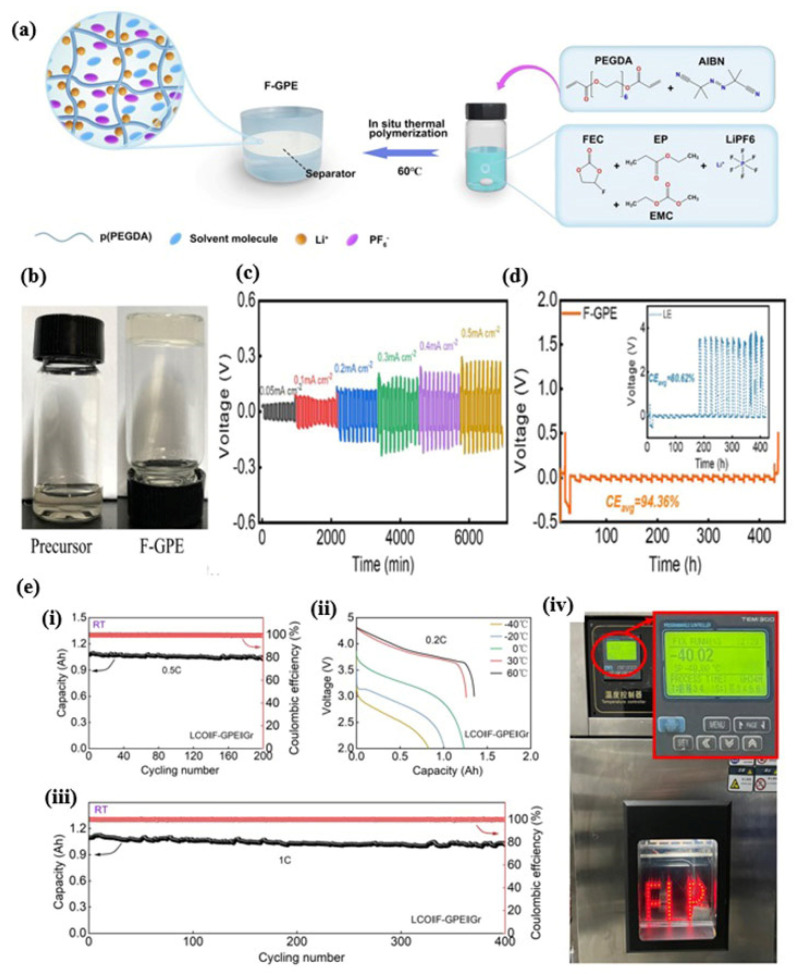
(**a**) Method for preparing F-GPE. (**b**) Visual representations of F-GPE. (**c**) Voltage–time profile for the plating/stripping process of a symmetric Li||F-GPE||Li cell at different current densities. (**d**) CEavg of Li||FGPE||Cu cell) at 0.5 mA cm^−2^. (**e**) (i) Cycling performance of LCO at room temperature. (ii) Discharge curves of LCO at different temperatures. (iii) Long-term cycling performance of LCO after 400 cycles at ambient temperature. (iv) Photo of LCO pouch battery lighting 70 LEDs at −40 °C; reproduced with permission from ref. [45], Elsevier.

H. He and colleagues conducted research on creating a highly effective fluorinated gel polymer electrolyte (F-GPE) using poly(ethylene glycol) diacrylate (PEGDA) as a crosslinking agent [45]. The process of preparing F-GPE by in situ thermal polymerization is shown in Figure 6a. The F-GPE displayed impressive electrochemical stability across an extensive voltage range of 2–5.6 V and maintained high ionic conductivity even at −40 °C. The optical image of F_GPE is shown in Figure 6b. This study emphasized the F-GPE’s capability to perform well at low temperatures while improving stability and electrochemical characteristics at elevated temperatures, making it a promising choice for large-scale lithium-ion battery applications across varying operating temperatures. In Figure 6c, the Li||F-GPE||Li cell showed stable overvoltage during Li stripping/plating at a current density of 0.5 mA cm^−2^, demonstrating remarkable interface compatibility of the GPE with Li metal. Figure 6d depicts the average coulombic efficiency of Li||Cu cells utilizing F-GPE and LE to assess Li plating/stripping behavior. The electrochemical performance of a complete battery with PEGDA-based electrolytes in LIBs was analyzed, revealing good cycling behavior with capacity retention rates of 95.3% after 200 cycles at 0.5 C and 92.9% after 400 cycles at 1 C, as seen in Figure 6e in section (i–iv). Herein, lithium cobalt oxide (LCO) was the testing electrode. 

### 3.5. Poly (4-Hydroxybutyl Acrylate) for Applications in Lithium-Ion Batteries

**Figure 7:**
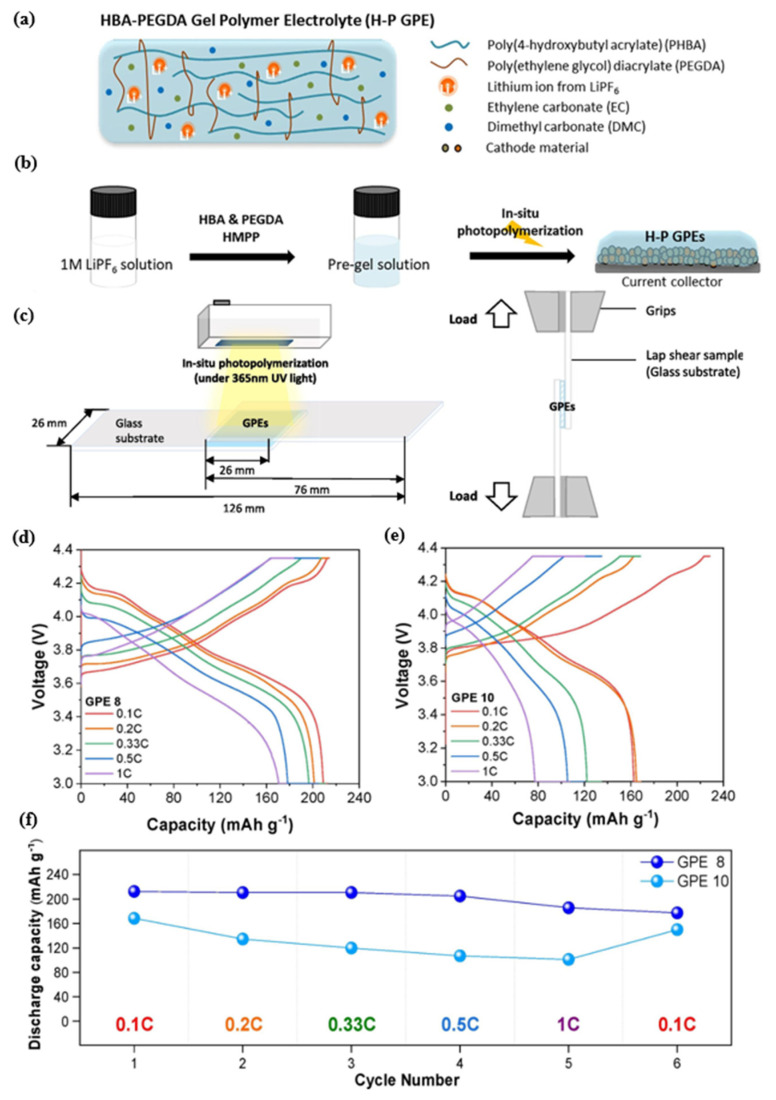
(**a**) Schematic diagram of the HP GPEs, which are innovative gel polymer electrolytes (H-P GPEs) derived from poly (4-hydroxybutyl acrylate) (PHBA). (**b**) Preparation of the H-P GPEs. (**c**) Adhesion properties of the H-P GPEs. (**c**) Schematic diagram illustrating the sample preparation procedure (**left**) and characterization (**right**) for the lap shear test. (**d**,**e**) Charge/discharge curves at different current rates of the electrochemical characteristics of GPE8 and GPE10 in half-cell battery configurations. (**f**) Rate cycling performance ranging from 0.1 C to 1 C; reproduced with permission from ref. [46], Elsevier.

Gel polymer electrolytes (GPEs) are known to enhance the safety and flexibility of lithium-ion batteries (LIBs). In this study, a poly (4-hydroxybutyl acrylate)-based GPE was introduced for its strong adhesion properties [46]. Sixteen GPE variants were created by adjusting liquid content, and monomer-to-crosslinker ratios yielded different types of gelation results: no gelation, formation of a viscous gel, and establishment of a stable gel (as shown in Figure 7a,b). The mechanical properties ranged from 0.92 kPa to 19.01 kPa. Adhesion tests revealed lap shear strength levels significantly higher than conventional GPEs (as shown in Figure 7c). The GPEs demonstrated noteworthy electrochemical stability, minimal current generation, efficient ionic conductivities, and displayed 99.69% coulombic efficiency, 212.37 mAh g^−1^ discharge capacity, and 87.43% capacity retention between 0.1 C and 1 C, as depicted in Figure 7d–f.

### 3.6. PEO Based GEPs

**Figure 8:**
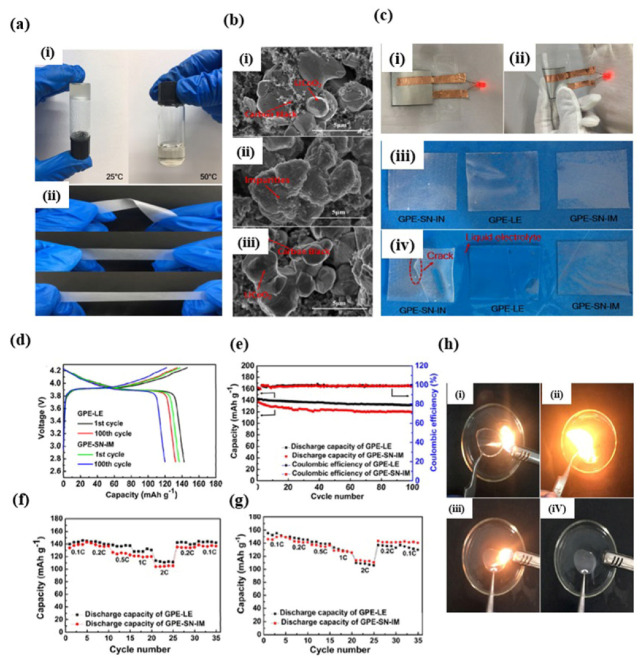
(**a**) (i) SN electrolytes at 25 °C and 50 °C, highlighting the change from a paste-like to a liquid state. (ii) Flexible and stretchable characteristics of the GPE-SN-IM film. (**b**) SEM images of the fresh LiCoO_2_ cathode and the cathodes from cells utilizing GPE-LE and GPE-SN-IM after 100 charge–discharge cycles at 55 °C, showing differences. (**c**) (i) Battery lighting up an LED with LiCoO_2_/GPE-SN-IM/Li_4_Ti_5_O_12_; (ii) GPE-SN-IM battery still powers LED after 100 folds; (iii) discharge capacities with different GPEs before folding; (iv) discharge capacities after 100 folds, GPE-LE shows electrolyte leakage. (**d**–**g**) Charge–discharge characteristics of LiCoO_2_/Li cells built with GPE-LE and GPE-SN-IM at a different condition. (**h**) Flame ignition tests for (i) GPE-LE and (iii) GPE-SN-IM. After contacting the flame, (ii) GPE-LE quickly burns but (iv) GPE-SNIM does not burn. Reprinted with permission from [48]. Copyright © 2018, American Chemical Society.

PEO was used as a polymer host along with a liquid electrolyte or GPE to create an electrolyte membrane that exhibited significant ionic conductivity, high lithium transference, and excellent thermal stability. An evaluation of the GPE’s electrochemical performance was carried out using a Li/GPE/LiFeO_4_ cell, showing reliable cycling stability and rate performance. The liquid electrolyte was enclosed within the solidified polymer structure, as illustrated in Figure 8a. Scanning electron microscope (SEM) images in Figure 8b (i–ii) display the prepared GPE as a flexible white membrane, indicating its potential application in the creation of wearable and other electronic devices [47]. The gel polymer electrolyte exhibited extended combustion duration without size alteration when exposed to fire, showcasing superior flame-retardant properties, as noted in Figure 8c in section (i–viii). This emphasizes the enhanced safety of the composite membrane over traditional separators, indicating its value in improving battery safety measures.

The development of new battery configurations integrating NCA/graphite GPE and NCA/graphite-Si/C GPE has shown promising advancements in performance and safety, evidenced by enhanced capacity retention post 200 cycles at 5 C, as shown in Figure 8d–g, discharge rate when compared with traditional liquid electrolyte batteries [49]. The unique structural design of GPEs is pivotal in facilitating the creation of a protective layer on the electrodes, thereby mitigating side reactions, boosting battery stability, and reducing gas generation. The introduction of PETEA-based GPE batteries has notably extended the cycle life (shown in Figure 8h), decreased gas emission, improved safety measures, and optimized electrode contact and resistance, collectively improving overall battery performance and safety standards in high-performance LIBs.

### 3.7. Succinonitrile-Based Gel Polymer Electrolyte

The study by Pengfei Lv et al. outlines the successful development of a durable gel polymer electrolyte–solid nanocomposite (GPE-SN) with enhanced mechanical strength, high ionic conductivity, and improved thermal stability for safer lithium-ion batteries [48]. This GPE-SN, created through a solution immersion method, showed resilience through 100 folding cycles without damage or loss in capacity, surpassing the performance of in situ polymerized films. Its superior cycling stability at high temperatures compared with traditional liquid electrolytes positions it as a promising choice for polymer electrolytes (Figure 8a,c). The discharge capacities and coulombic efficiencies of LiCoO_2_-based half cells utilizing GPE-LE/Li and GPE-SN-IM/Li at 25 °C and 55 °C (SEM image shown in Figure 8b) are discussed, highlighting performance variations over 100 charge–discharge cycles, as shown in Figure 8d–g. Flammability is a crucial safety aspect for battery polymer electrolytes. Results from an ignition test (as shown in Figure 8h) compare GPEs containing SN and EC/DMC. While GPE-LE with EC/DMC ignites upon flame exposure, GPE-SN-IM produces minimal smoke, indicating lower flammability. This characteristic positions GPE-SN-IM as a safer alternative for lithium-ion batteries 

## 4. Application of Lithium-Ion Battery in Long Cycle Life Span

**Figure 9:**
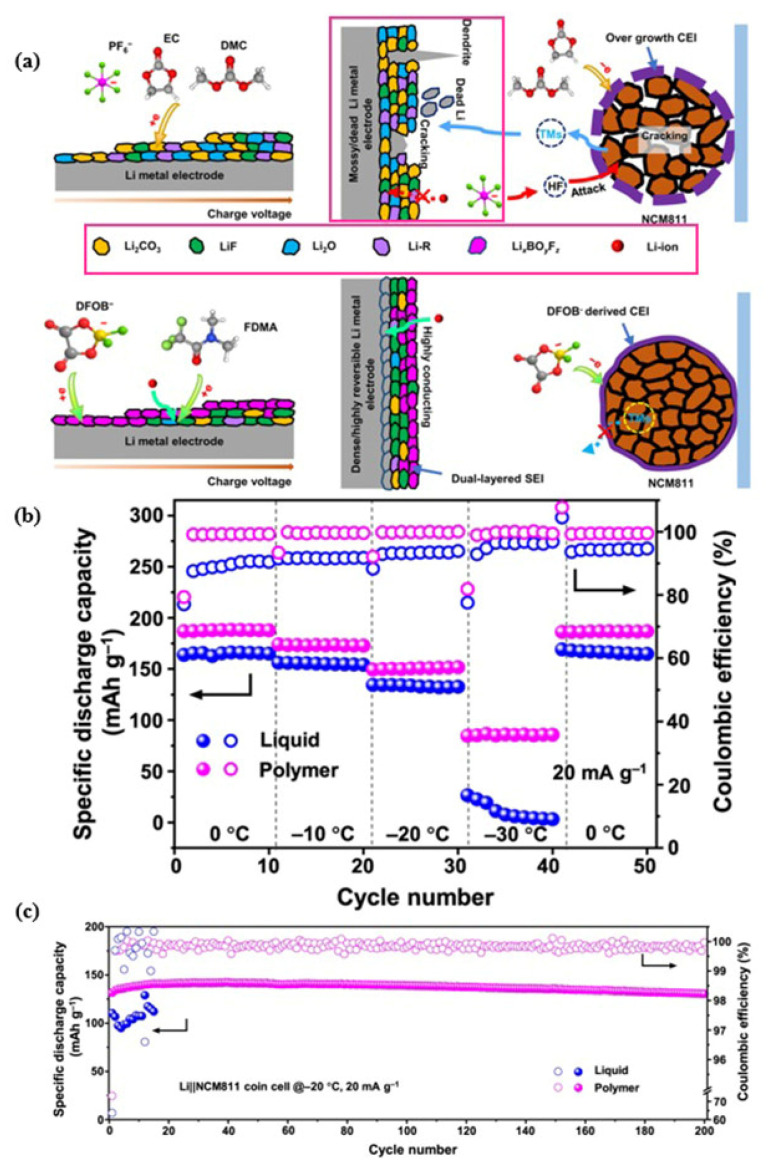
(**a**) (i) Diagram of the SEI layer on a lithium metal electrode (**left**) and its degradation in a Li||NCM811 cell (**right**) with a carbonate electrolyte. (ii) Diagram of the SEI layer on a lithium metal electrode (**left**) and prevention of degradation in a Li||NCM811 cell (**right**) with the polymer electrolyte from this study. The charge voltage shows the cell voltage during charging. (**b**) Discharge capacities along with coulombic efficiencies (CEs) of both electrolytes in Li||NCM811 coin cells at various temperatures and a rate of 20 mA g^−1^. (**c**) Cycling performance of both electrolytes in Li||NCM811 coin cells at −20 °C and a rate of 20 mA g^−1^, adopted from ref. [50], Springer Nature.

Z. Li and team recently reported on a new solid-state polymer electrolyte with remarkable ionic conductivity even at −20 °C. This electrolyte is created through in situ polymerization using a 1,3,5-trioxane-based precursor [50]. Utilizing this polymer electrolyte results in the formation of a two-layer solid electrolyte interphase on the lithium metal electrode, which stabilizes the positive electrode made of LiNi_0.8_Co_0.1_Mn_0.1_O_2_. The improved charge transfer at lower temperatures inhibits dendrite formation on the lithium metal electrode, enabling reliable operation of Li||LiNi_0.8_Co_0.1_Mn_0.1_O_2_ coin and pouch cells down to −30 °C. Notably, a Li||LiNi_0.8_Co_0.1_Mn_0.1_O_2_ coin cell cycled successfully at −20 °C and 20 mA g^−1^, maintaining over 75% (about 151 mAh g^−1^) of its initial discharge capacity after cycling at 30 °C with the same current density (as depicted in Figure 9b,c). Conversely, while liquid organic electrolytes in lithium-ion batteries can cause issues like inadequate ionic conduction and dendrite growth, the use of fluoroethylene carbonate (FDMA) containing electron-withdrawing groups assists in developing a favorable SEI layer rich in LiF and lacking Li_2_CO_3_, as depicted in Figure 9a.

## 5. Application Gel Polymer Electrolytes for Several Beneficial Properties

General requirements of GPEs. In LIBs, gel polymer electrolytes (GPEs) are positioned between the cathode and anode (such as lithium metal, graphite, etc.), serving dual roles as electrolytes and separators. As a result, GPEs have a significant influence on the overall performance of LIBs. Given their vital function in the LIB setup, GPEs are expected to exhibit specific essential properties [51].

### 5.1. Thermal and Chemical Stability

The stability of gel polymer electrolytes (GPEs) is crucial to prevent unwanted chemical reactions upon contact with electrodes. While GPEs can offer comparable ionic conductivity to liquid electrolytes, the presence of embedded solvents may lead to irreversible capacity loss due to passivation layer formation on electrode surfaces. To address this issue, the use of electrolyte additives and stable lithium salts can be effective [52,53,54]. GPEs combine polymer matrices with organic liquids as plasticizers, underscoring the importance of polymer metrics in preserving thermal stability. Therefore, a thermally robust polymer is essential for GPE systems [55,56,57]. Instead of relying solely on a single polymer chain, employing a composite polymer structure can substantially improve the overall thermal stability of GPEs [58].

### 5.2. Improved Interfacial Stability

Enhancing the interfacial stability of lithium-ion batteries, gel polymer electrolytes have emerged as a pivotal solution, offering a protective layer at the interface between the electrode and electrolyte to inhibit dendrite formation and minimize irreversible reactions at the electrode surface, subsequently extending cycle life and improving charge/discharge efficiency. In a comprehensive study, the crucial role of gel polymer electrolytes in creating a stable interphase was underscored, showcasing their ability to mitigate dendrite growth and enhance the overall efficiency of lithium-ion batteries [59]. The research demonstrated how gel polymer electrolytes foster a robust interfacial environment, reducing the risk of short circuits and improving the long-term reliability of battery systems. Furthermore, in-depth investigations were conducted into the interfacial stability benefits of gel polymer electrolytes, highlighting their ability to decrease capacity fading, enhance electrochemical performance, and prolong battery lifespan [60]. These findings emphasize the profound impact of gel polymer electrolytes on interfacial stability in lithium-ion batteries, advancing the understanding of how these electrolytes contribute to enhanced battery performance and safety. Notably, the thermal management capabilities of lithium-ion batteries are also bolstered by gel polymer electrolytes through their promotion of efficient heat dissipation, ensuring the operational safety of battery systems across a wide range of temperature fluctuations. This collective body of research underscores the substantial role of gel polymer electrolytes in improving interfacial stability, reliability, and efficiency in lithium-ion battery technology, setting a precedent for further advancements in energy storage applications.

### 5.3. Wide Operating Temperature Range

Significant attention has been garnered in lithium-ion battery research towards gel polymer electrolytes due to their wide operating temperature range, positioning them as a focal point for enhancing battery performance and safety across different environmental conditions. Unlike traditional liquid electrolytes, improved thermal stability is exhibited by gel polymer electrolytes, thereby reducing the hazards related to thermal runaway events and enhancing the overall safety profile of battery systems. Recent studies have highlighted the exceptional thermal management capabilities of gel polymer electrolytes, showcasing their ability to withstand high-temperature conditions without compromising performance [61]. Moreover, the reduced flammability of gel polymer electrolytes is a critical safety feature that minimizes the risks of fire hazards, especially in applications where safety is paramount. Their non-flammable or less flammable nature helps contain potential fire incidents within the battery system, preventing the spread of flames and ensuring safe operation in a variety of settings. The mechanical robustness of gel polymer electrolytes is another key advantage, providing flexibility and resilience to withstand physical stresses and prevent electrolyte leakage. One study demonstrated the superior mechanical properties of gel polymer electrolytes, highlighting their ability to maintain structural integrity and enhance the reliability of battery systems under challenging conditions [62]. Overall, the wide operating temperature range, improved thermal stability, reduced flammability, and mechanical robustness of gel polymer electrolytes are collectively contributing to their suitability for diverse lithium-ion battery applications. Continued advancements in gel polymer electrolyte research are key to further optimizing these properties, ensuring the continued progress of battery technology towards safer, more reliable, and high-performance energy storage solutions.

### 5.4. Mechanical Stability

Gel polymer electrolytes (GPEs) play a vital dual role within lithium-ion batteries (LIBs) as they act both as a conductive pathway between electrodes and as a separator. The suitability of GPEs in LIBs is heavily reliant on the mechanical attributes of the polymers employed [22,63,64]. However, standard GPEs derived from plasticized linear polymer chains frequently exhibit inadequate mechanical properties, creating a challenge in finding a balance in the relationship between ionic conductivity and mechanical robustness due to the presence of organic liquids within these systems. A potential solution lies in integrating polymer chains containing crosslinkable components into GPE structures [56]. Another avenue involves introducing a three-phase architecture involving a polymer network and an inorganic salt solution to bolster the mechanical integrity of GPEs.

### 5.5. Electrochemical Stability

The electrochemical durability of gel polymer electrolytes (GPEs) is essential as it influences the permissible operational voltage range of lithium-ion batteries (LIBs) during charging and discharging. GPE materials must remain unreactive towards both the cathode and anode electrodes, requiring their oxidation potential to exceed the lithium-ion embedding potential in the cathode and their reduction potential to be lower than that of lithium metal in the anode [63]. Given that LIBs typically function within voltage ranges from 0 to 4 V vs. Li/Li^+^ (occasionally extending to around 5 V), GPE materials must demonstrate electrochemical stability within this voltage window to establish a thermodynamically steadfast system [52,54,57].

**Figure 10:**
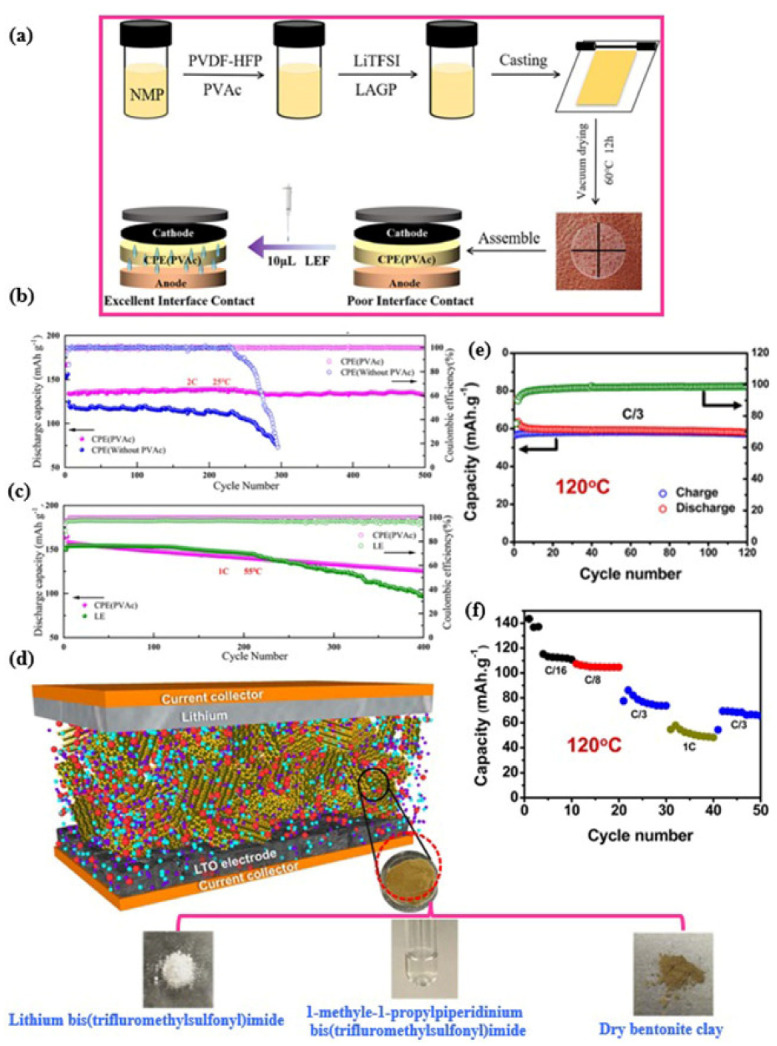
(**a**) Schematic of the preparation process for CPE(PVAc) and its battery configuration. (**b**) Comparison of long-term cycling performance between LiFePO_4_|CPE(PVAc)|Li and LiFePO_4_|CPE (without PVAc)|Li at a rate of 2 C and a temperature of 25 °C. (**c**) Long-term cycling performance comparison of LiFePO4|CPE(PVAc)|Li and LiFePO_4_|LE|Li at a rate of 1 C and 55 °C, reproduced with permission from ref. [65], Elsevier. (**d**) Components used to create the clay–RTIL quasi-solid-state electrolyte and the structure of the LIB. (**e**) Cyclic stability and rate capability of Li_4_Ti_5_O_12_ half cells at 120 °C. (**f**) LTO half-cells were evaluated at 120 °C with composite electrolytes; reprinted with permission from [66]. Copyright © 2015, American Chemical Society.

Researchers led by Dakun Song developed a rigid/flexible gel polymer electrolyte (GPE) labeled CPE(PVAc). This GPE, comprising PVDF-HFP/LAGP/PVAc/LiTFSI, was created using a straightforward solution casting technique (as shown in Figure 10a). The technique facilitated the creation of a stable solid electrolyte interphase (SEI) layer rich in LiF on the lithium anode, effectively preventing the growth of lithium dendrites. In lithium coin cells containing LiFePO_4_|CPE(PVAc)|Li, exceptional initial discharge specific capacities were achieved, with 135.4 mAh g^−1^ at 2 C and 25 °C, and 157.5 mAh g^−1^ at 1 C and 55 °C. Moreover, these cells exhibited impressive long-term cycling stability, maintaining 97.6% capacity after 500 cycles at 2 C @25 °C, and 80% capacity retention after 400 cycles at 1C @55 °C (as shown in Figure 10b,c) [65]. A newly designed quasi-solid-state electrolyte for high-temperature lithium-ion batteries has been introduced, combining the solidity of a solid structure with the fluidity of a liquid. This innovative electrolyte formulation consists of micro flakes of clay particles suspended in a solution of lithium-based room temperature ionic liquid, forming a quasi-solid system that maintains structural integrity even at temperatures up to 355 °C. Testing with a rechargeable lithium battery incorporating a Li_4_Ti_5_O_12_ (LTO) electrode demonstrated consistent capacity retention over 120 cycles (refer to Figure 10d). Figure 10e,f visualize the components employed in creating the clay−RTIL quasi-solid-state electrolyte and illustrate the envisioned structure of a lithium-ion battery [66].

**Figure 11:**
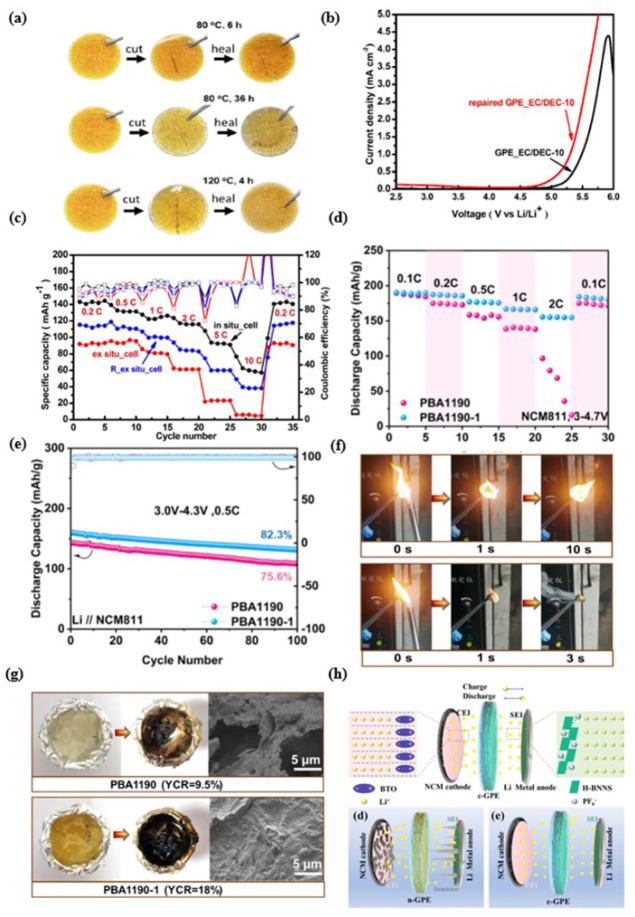
(**a**) Self-healing of GPE_EC/DEC-10 under different thermal treatments. (**b**) Comparison of linear sweep voltammetry curves before and after a cut/self-healing process in GPE_EC/DEC-10. (**c**) Cycling at various C rates, adopted from ref. [67]. (**d**) Rate performance with PBA1190 and PBA1190-1. (**e**) Cycle performance with PBA1190 and PBA1190-1 in the voltage range at 0.5 C for 100 cycles (**f**) Combustion tests of PBA1190 (i) and PBA1190-1 (ii) electrolytes in air. (**g**) Images of residual char following the thermal oxidation degradation of PBA1190 (i) and PBA1190-1 (ii) electrolytes at 500 °C for 0.5 h, reproduced with permission from ref. [68], Elsevier. (**h**) HRTEM analyses of NCM-523 cathodes from Li/NCM-523 cells with n-GPE and c-GPE-2 after 30 cycles at 0.5 C. It also includes schematic diagrams of batteries with c-GPE, and with n-GPE and c-GPE, reproduced with permission from ref. [69], Elsevier.

W.-T. Tsai et al. introduced dissociative covalent adaptable networks (CANs) into gel polymer electrolytes (GPEs) to enhance self-healing properties, thus improving cycling stability in LIBs. The GPE, created through in situ crosslinking of TF and TMI with liquid electrolytes, exhibited amine and hydroxyl groups to boost compatibility with liquid electrolytes and increase their content within the GPE. The LIB cells demonstrated autonomous capacity recovery during cycling, showcasing prolonged stability over 500 cycles at 2 C with minimal capacity loss decline at a high charging rate of 10 C, showcasing complete recovery of specific capacity across the operation range from 10 C down to 0.2 C (as shown in Figure 11c) attributed to the self-healing capability enabled by the dissociative CANs formed by TF and TMI (as shown in Figure 11a,b) [67].

## 6. Application of Gel Polymer Electrolyte in High-Nickel Cathode

Gel polymer electrolytes (GPEs) present numerous advantages over traditional liquid organic electrolytes, particularly in battery applications. One of their key strengths lies in their enhanced safety features, as the semi-solid nature of GPEs reduces the risks of leakage and flammability associated with liquid electrolytes, promoting device safety [70]. Additionally, the gel structure of GPEs provides superior mechanical stability, ensuring structural integrity even under physical stress, leading to enhanced battery longevity and reliability. Moreover, GPEs exhibit lower volatility compared with liquid electrolytes, extending battery lifespan and enhancing thermal stability across various temperature ranges. The ability of GPEs to operate at higher voltages benefits high-energy applications by improving energy density and overall performance. Furthermore, GPEs display increased resistance to electrolyte decomposition, maintaining ionic conductivity for efficient energy storage and transfer [71]. This combination of high ionic conductivity and mechanical robustness presents GPEs as a balanced alternative to liquid organic electrolytes, addressing historical safety and performance concerns associated with conventional systems. However, challenges exist with GPEs that require attention. These include lower ionic conductivity compared with liquid electrolytes, potentially affecting battery performance, especially in colder temperatures. The intricate and costly production processes of GPEs, involving precise polymerization and the incorporation of ionic salts and plasticizers, lead to higher manufacturing expenses. Limited compatibility with certain electrode materials may compromise the stability of the electrode/electrolyte boundary, with impact on long-term battery performance. Despite offering improved mechanical stability, GPEs can experience dehydration or swelling under extreme conditions, potentially affecting functionality and lifespan. Additionally, the relatively slower ion transport in GPEs compared with liquid electrolytes can limit rate capability and influence battery power performance. Nonetheless, ongoing research and development initiatives are dedicated to overcoming these challenges to enhance the performance and commercial viability of gel polymer electrolytes [72].

### 6.1. Application in High-Performance Cathode 

Gel polymer electrolytes (GPEs) are extensively utilized in lithium-ion batteries due to their enhanced safety, mechanical stability, and flexibility. They reduce risks of leakage and flammability compared with liquid electrolytes and enhance energy density with higher voltage operation. GPEs ensure efficient energy storage and transfer, making them ideal for consumer electronics, electric vehicles, and renewable energy storage systems. In modern lithium-ion batteries utilizing high-nickel cathodes like NCM and NCA, and materials such as LiFePO_4_, GPEs are increasingly preferred over traditional liquid electrolytes due to their myriad advantages [43,50,73]. High-nickel cathodes boast exceptional energy density and capacity, making them crucial components for high-performance applications in electric vehicles and advanced electronics. LiFePO_4_, known for its outstanding thermal stability, extended cycle life, and safety features, is ideal for applications prioritizing robust and consistent performance. However, despite their benefits, these materials face challenges related to safety issues, thermal instability, and accelerated degradation, which GPEs can effectively address. GPEs offer significant safety enhancements over liquid electrolytes by minimizing risks of leakage and flammability due to their semi-solid composition, bolstering overall safety. They also deliver enhanced mechanical stability, which helps mitigate volume changes and mechanical strains experienced during charge–discharge cycles by high-nickel cathodes and LiFePO_4_, thereby preserving battery integrity. Additionally, GPEs support the high-voltage operations characteristic of NCM and NCA cathodes while being compatible with the lower voltage range of LiFePO_4_, crucial for optimizing energy density and effectiveness across diverse battery chemistries. Significant improvements in polymer matrix technologies, such as PVDF, PAN, and PEO, have greatly enhanced the ionic conductivity of GPEs [30,31,74]. These polymers, paired with lithium salts like LiPF_6_, LiBF_4_, and LiTFSI, create conductive and stable gels that prolong battery cycling life. Furthermore, GPEs form a more stable solid-electrolyte interphase (SEI) with high-nickel cathodes and LiFePO_4_ compared with liquid electrolytes, reducing electrolyte degradation and cathode damage to enhance battery longevity. Despite the initial higher costs and synthesis complexities of GPEs, their long-term advantages, including enhanced safety, improved thermal stability, and extended operational life, position them as a compelling alternative. Ongoing research endeavors are continuously addressing these challenges, making GPEs a more attractive option for high-energy applications demanding enhanced performance and dependability. Thus, GPEs represent a significant innovation in creating safer, more efficient, and longer-lasting lithium-ion batteries utilizing high-nickel and LiFePO_4_ cathodes [66,75].

### 6.2. NCA and NCM Cathode

The flowable liquid polymer electrolyte (LPE) effectively coats and infiltrates the electrode, promoting excellent interfacial contact and enabling LiNi_0.8_Co_0.1_Mn_0.1_O_2_ (NCM811) batteries to sustain stable long-term cycling at higher temperatures up to 120 °C with approximately 100% coulombic efficiency. Enhancing the electrolyte’s surface coverage and intrinsic stability is crucial for establishing a stable interface [76]. Creating a flexible interface between the electrolyte and the electrode is vital for optimal battery performance. In their research, Wang and the team utilized a poly(acrylonitrile-butadiene) (PAB) polymer coating on the LiNi_0.6_Mn_0.2_Co_0.2_O_2_ cathode material. This nano-soft PAB layer notably enhanced physical interaction between the cathode and the solid electrolyte. When combined with lithium metal and a poly (ethylene-acrylic ester) (PEA) solid electrolyte, the resulting solid-state battery exhibited exceptional rate performance stability, retaining 75% of its capacity after 400 cycles [77]. Performance of cathode is presented in Table 2.

### 6.3. Application of GPEs in High-Nickel Cathode to Enhance Safety

Gel polymer electrolytes are emerging as a preferred choice in lithium-ion battery technology due to their superior safety attributes compared with traditional liquid electrolytes. Safety is paramount in battery applications, especially as lithium-ion batteries power various devices ranging from smartphones to electric vehicles [90]. Gel polymer electrolytes offer several key safety benefits that make them a compelling option for battery systems. One significant safety advantage of gel polymer electrolytes is their enhanced thermal stability. Unlike conventional liquid electrolytes, which can be prone to thermal runaway at high temperatures, gel polymer electrolytes are designed to be more thermally stable, reducing the risk of safety hazards like fires or explosions. The gel structure in these electrolytes provides a stable environment for ion transport, lowering the likelihood of thermal decomposition. Moreover, the lower flammability of gel polymer electrolytes contributes to the overall safety of lithium-ion batteries. In case of malfunctions or accidents, the non-flammable or less flammable nature of gel electrolytes helps contain potential fire risks within the battery, preventing fire spread. This feature is crucial for ensuring safe battery operation across various applications [91].

Another important safety aspect of gel polymer electrolytes is their mechanical strength. The gel-like consistency offers flexibility and resilience, enabling the electrolyte to endure physical stresses and prevent leaks. This mechanical robustness enhances the battery’s structural integrity, decreasing the chances of electrolyte exposure and associated risks [92]. The safety advantages of gel polymer electrolytes make them an attractive choice for lithium-ion battery applications demanding high safety standards, like electric vehicles and aerospace technologies. By mitigating risks related to thermal runaway, flammability, and mechanical issues, gel polymer electrolytes enhance the reliability and safety of battery systems. As battery technology continues to progress, the development of gel polymer electrolytes with enhanced safety features signifies a significant advancement. Future research endeavors may focus on optimizing gel electrolyte formulations and properties to meet the evolving requirements for safe high-performance lithium-ion batteries in various sectors. In summary, the improved safety characteristics of gel polymer electrolytes can potentially transform the landscape of lithium-ion battery technology, offering a safer and more dependable energy storage solution for a broad range of applications. By addressing critical safety concerns and reducing risks, gel polymer electrolytes play a pivotal role in elevating the safety standards of battery systems in today’s dynamic technological environment [93]. C. Wang and colleagues devised a dual-functional flame retardant (HCCP-TMP), merging hindered amine and cyclophosphazene to address both flame resistance and free radical scavenging. By incorporating just 1 wt% of HCCP-TMP, a polyacrylate-based GPE acquires non-combustibility. NCM811//graphite pouch cells coupled with this GPE exhibit resilience against combustion and mechanical stress. The NCM811/Li battery utilizing this non-flammable GPE demonstrates exceptional high-voltage cycling, with 82.2% capacity retention after 100 cycles at 2 C within 3.0–4.7 V, showcasing its robust performance (as shown in Figure 11d,e) [48,68,69,94]. In a separate investigation, a research initiative aimed to stabilize glymes at highly oxidizing electrode potentials by engineering self-limiting cathode electrolyte interfaces (CEIs) to elevate lithium-ion battery cathode efficiency and ensure the robustness of polymer electrolytes in high-voltage contexts, ultimately amplifying battery safety and efficacy [94]. In the absence of HCCP-TMP, PBA1190 underwent complete combustion post-flame exposure. In contrast, PBA1190-1 self-extinguished within 3 s, showcasing significant flame-retardant properties (as shown in Figure 11f). Residual char from the thermal degradation of PBA1190 and PBA1190-1 at 500 °C was collected. Refer to Figure 11g for digital and SEM images of both materials before and after combustion. The novel design of a multifunctional gel polymer electrolyte (GPE) effectively tackles issues related to lithium dendrite growth and cathode stability. By integrating barium titanate (BTO) and hexagonal boron nitride nanosheets (h-BNNS) into a polyvinylidene fluoride-hexafluoropropylene (PVDF-HFP) matrix using electrospinning, the GPE demonstrated impressive performance. The Li/NCM-523 battery exhibited excellent rate capability and cycling stability, maintaining 80% capacity after 170 cycles at 0.5 C. Similarly, the Li/NCM-811 battery sustained 81% capacity after 100 cycles at 0.5 C. Different GPE variations, such as n-GPE, bto-GPE, and c-GPE-1 to c-GPE-3, were created through immersion in a liquid electrolyte solution. Testing indicated that the Li/c-GPE-3/Li cell showed stable voltage over 310 h, highlighting effective dendrite suppression and interface stability provided by c-GPE-2 (as shown in Figure 11h) [69].

**Figure 12:**
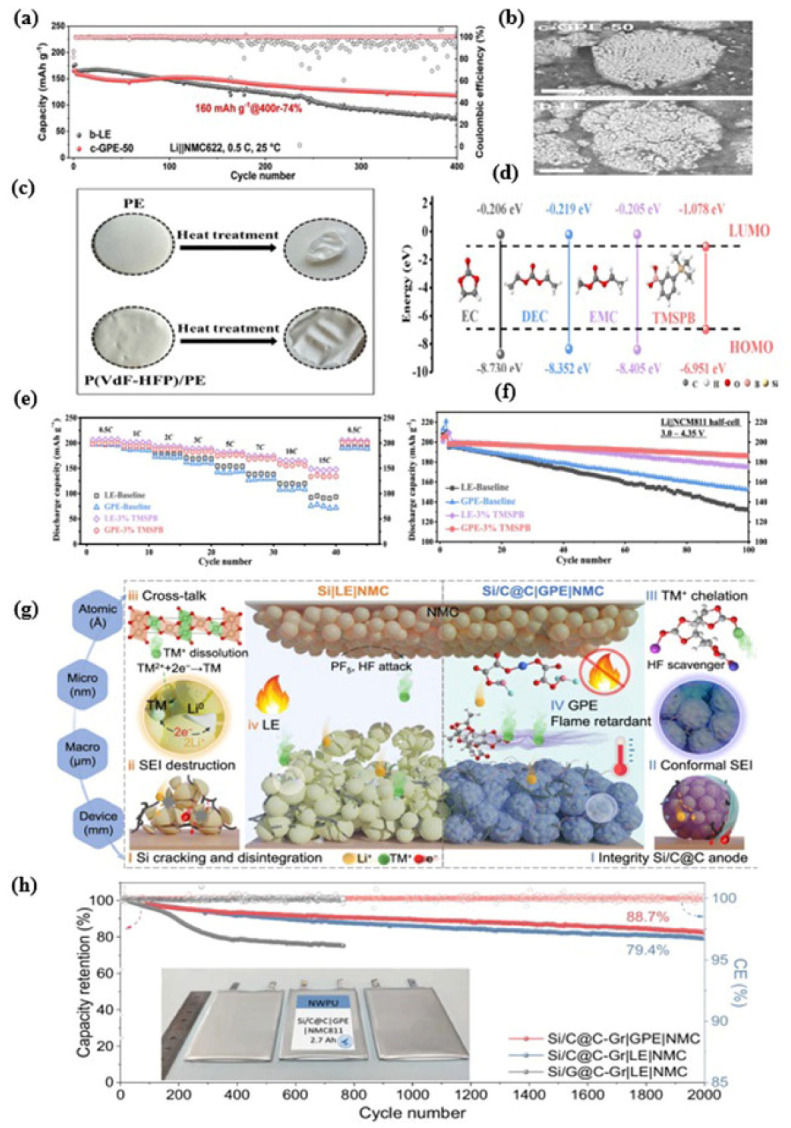
(**a**) Cycling performances of the Li||NMC622 batteries with b-LE and c-GPE-50 at 0.5 C. (**b**) SEM images, reproduced with permission from ref. [95], Elsevier. (**c**) Optical images of PE and (PVDF-HFP)/PE membranes before and after 30 min heat treatment at 135 °C. (**d**) Optimized structures and LUMO/HOMO energies of carbonate solvents (EC, DEC, EMC) in liquid electrolyte and TMSPB additive. (**e**) Cycling of Li||NCM811 half-cells with various electrolytes at different charge rates in the voltage range of 3.0–4.35 V. (**f**) Rate capability of Li||NCM811 cells in different electrolytes; reprinted with permission from [98]. Copyright © 2022, American Chemical Society. (**g**) Si-based anode||NMC full cell model, showing the fading mechanisms in the Si|LE|NMC prototype, including Si cracking, Li^+^ depletion, SEI rupture, and safety hazards. It also highlights the features of the Si/C@C|GPE|NMC system, emphasizing the Si/C@C anode design with improved interfacial stability, suppressed crosstalk effect, and flame retardancy of the GPE. (**h**) Electrochemical performance of the Si/C@C-Gr|GPE|NMC full cell, focusing on the cycling behavior of the cells, adopted from ref. [99], Springer Nature.

Recent studies have delved into utilizing a gel polymer electrolyte (GPE) to address crack propagation issues in high-nickel cathodes [95]. The research focused on c-GPE-50, known for its exceptional oxidative stability, in combination with a high-voltage cathode (NMC622) to develop a high-energy-density battery system. In the Li|c-GPE-50|NMC622 configuration, an impressive discharge capacity of 160 mAh g^−1^ at 0.5 C was achieved. Notably, after 300 and 400 cycles, this setup demonstrated outstanding cycling performance, retaining 80% and 74% of its capacity, respectively. This outperformed the Li|b-LE|NMC622 battery utilizing liquid electrolyte, which retained only 45% capacity after 400 cycles (as shown in Figure 12a). Examination of the cycled NMC622 particles from the Li|c-GPE-50|NMC622 battery showcased well-preserved mechanical integrity without visible cracks (as shown in Figure 12b), unlike the cracked particles in the Li|b-LE|NMC622 battery. This indicates improved stability and decreased electrolyte consumption in the c-GPE-50 system [96,97].

In a recent study, a new fluorinated phosphate crosslinker was introduced for application in gel polymer electrolytes in high-voltage lithium metal batteries (LMBs), achieving remarkable electrochemical performance alongside high safety standards. By integrating this fluorinated phosphate crosslinker (FP) into the GPE, LMBs exhibited consistent cycling behavior even at a high cut-off voltage of 4.6 V (versus Li/Li^+^) with various high-voltage cathode materials. Particularly, the LiNi_0.6_Co_0.2_Mn_0.2_O_2_ battery utilizing FP-GPE demonstrated an exceptionally long cycle life of 1200 cycles, maintaining an impressive capacity retention rate of 80.1% [100]. The introduction of the innovative boron compound, 3-(trimethylsilyl)-phenylboronic acid (TMSPB), as a synergistic additive within a (PVDF-HFP) based gel polymer electrolyte (GPE) has transformed the development of an enhanced GPE setup. In Figure 12c, a visual comparison highlights the thermal performance difference between a polyethylene (PE) separator and a (PVDF-HFP)/PE membrane after exposure to 135 °C for 30 min. The PE separator’s shrinkage due to polymer melt emphasizes its limited thermal endurance, while the structural robustness of the (PVDF-HFP)/PE membrane remains unaffected, showcasing the strong mechanical integrity of the (PVDF-HFP) copolymer. Timely oxidation shields against electrolyte breakdown, creating a barrier between the electrolyte and NCM811 cathode (Figure 12d). With TMSPB-enriched GPE configurations like GPE-baseline, LE-3% TMSPB, and GPE-3% TMSPB, capacity retentions of 78%, 88%, and 94% (Figure 12e) highlight the additive’s effectiveness. Notably, NCM811 in GPE-TMSPB achieves a capacity of 133.5 mAh g^−1^ at 15 C (Figure 12f), doubling capacity versus GPE-baseline [98].

A recent groundbreaking discovery involves proposing a gel polymer electrolyte for the NMC prototype to enhance the Si anode’s mechanical integrity and stabilize interfacial properties with transitional cations (Figure 12g). Through integrating conformal gel polymer electrolyte encapsulation with spatially arranged Si anode and NMC811 cathode, a 2.7 Ah pouch-format cell has achieved impressive results: high energy density of 325.9 Wh kg^−1^ (based on the entire pouch cell), 88.7% capacity retention over 2000 cycles (Figure 12h), self-extinguishing capability, and broad temperature tolerance. This breakthrough also utilizes an in situ polymerization strategy for gel polymers involves initiating polymerization directly within the solution to form the gel electrolytes in Si||Ni-rich lithium-ion batteries [99].

**Figure 13:**
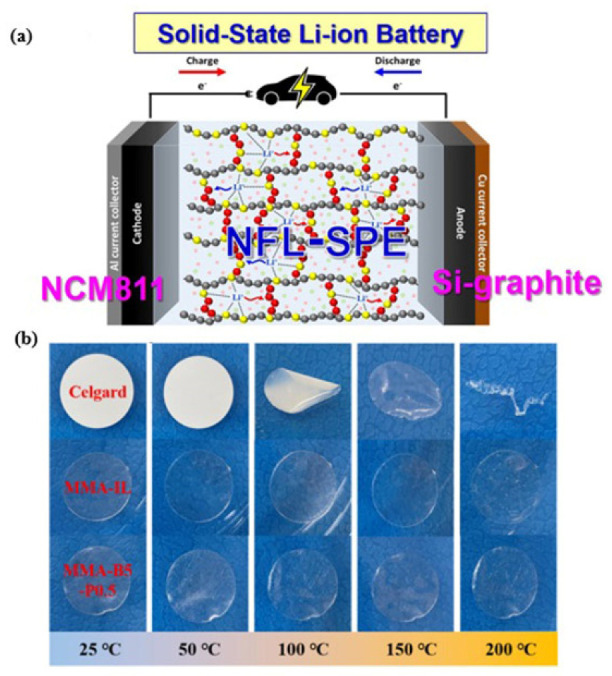
(**a**) Schematic of a Si-graphite/NFL-SPE/NCM811 solid-state Li-ion full cell, reproduced with permission from ref. [101], Elsevier. (**b**) Images depicting thermal shrinkage for the Celgard separator, MMA-IL, and MMA-B5-P0.5, reproduced with permission from ref. [102], Elsevier.

In a study involving a combination of NFL-SPE and porous Si-graphite active material particles, similar to the scenario outlined for NCM811 earlier (as shown in Figure 13a), a solid-state Si-graphite/NFL-SPE/NCM811 coin full cell was assembled utilizing NFL-SPE [101].

### 6.4. Application of LiFePO_4_ Cathode for Flame Safety

**Figure 14:**
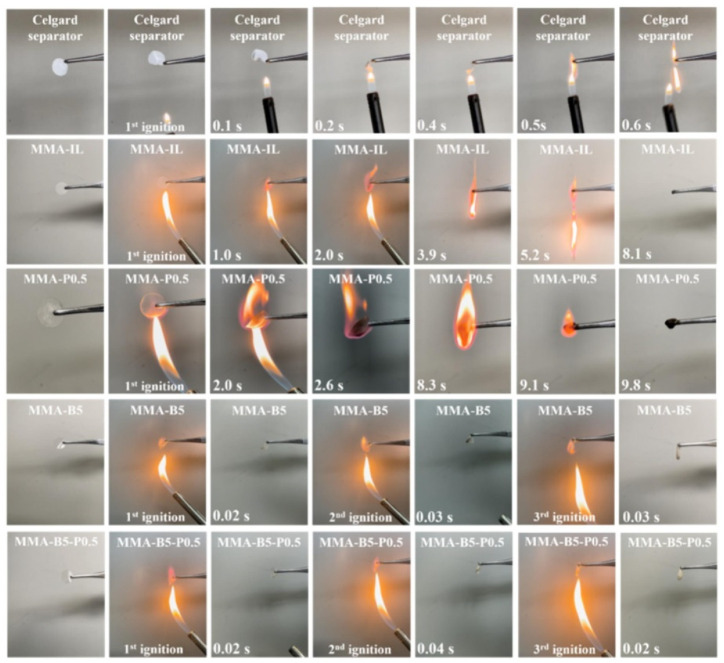
Fire resistance tests for the Celgard separator and GPE films, reproduced with permission from ref. [102], Elsevier.

In 2024, Y. Du and colleagues introduced a cutting-edge non-flammable gel polymer electrolyte (GPE) featuring a 3D interpenetrating network designed for high-performance lithium-ion batteries [102]. The batteries based on this composite GPE, including LiFePO_4_||Li, NCM523||Li, and LiFePO_4_||graphite configurations, demonstrated excellent cyclic stability at a 1 C rate. Testing on pouch cells utilizing this GPE showcased remarkable non-flammable properties and enhanced safety through nail penetration and ignition tests, resulting in a notable reduction of the maximum surface temperature by 63.3%. Furthermore, an evaluation of the dimensional stability of separators such as Celgard, MMA-IL, and MMA-B5-P0.5 when subjected to thermal exposure ranging from 25 °C to 200 °C provided valuable findings. The Celgard separator exhibited significant shrinkage beyond 100 °C and exhibited signs of near melting at 200 °C after exposure for 30 min at 100 °C, indicating inadequate thermal stability (as shown in Figure 13b). Subsequent analysis in a different figure highlighted the rapid shrinkage of the Celgard separator without direct flame contact, its propensity for easy ignition, intense burning, and production of melting droplets, emphasizing the critical need for separators with enhanced thermal and fireproof characteristics in ensuring safety in energy storage systems (as shown in Figure 14). Schematic of a Si-graphite/NFL-SPE/NCM811 solid-state Li-ion full cell is shown in Figure 13a.

In 2022, scientists developed two variants of solid gel polymer electrolytes (SGPEs)—HA-BF_4_ and HA-TFSI. These SGPEs were created by blending chitosan-based polymer substrates with ionic liquids for lithium metal batteries. The most effective SGPE film, HA-BF4-80, exhibited remarkable high-temperature stability up to 270 °C, high ionic conductivity measuring 2.03 × 10^−3^ S cm^−1^ at 35 °C, along with outstanding interface stability and electrode compatibility [103,104,105]. A new gel polymer electrolyte (GPE) utilizing poly (vinylidene fluoride-co-hexafluoropropylene) (PVDF-HFP) with an uneven structure has been innovated to effectively suppress lithium dendrite growth. This advanced asymmetric GPE offers high lithium-ion conductivity. When applied in Li|Li symmetric cells, this superior GPE shows significantly enhanced cycling stability compared with those using traditional liquid electrolytes. Furthermore, Li|LiFePO_4_ batteries incorporating the asymmetric GPE exhibit exceptional electrochemical performance, achieving a remarkable coulombic efficiency of 99.5% at a 2 C rate after 600 cycles [106].

### 6.5. The Overview of Challenges for the Applications of GPEs in LIBs

**Scheme 5:**
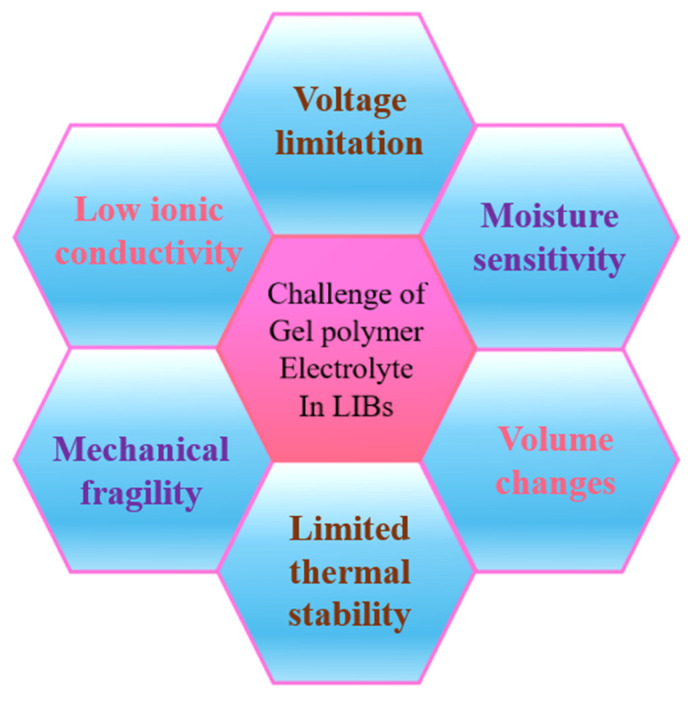
Overview of challenges for the applications of GPEs in LIBs.

The application of gel polymer electrolytes (GPEs) in lithium-ion batteries faces several key challenges that must be addressed for effective and widespread use. The overview of challenges for the applications of GPEs in LIBs has been shown in Scheme 5. Firstly, achieving high ionic conductivity comparable to liquid electrolytes is crucial, as inefficiencies here can limit battery performance; this can be targeted through the incorporation of ionic liquids or plasticizers. Ensuring mechanical stability is also essential, as GPEs must retain structural integrity across numerous charge–discharge cycles and varying temperatures, which can be improved by introducing reinforcing fillers or crosslinking techniques. Additionally, electrochemical stability is critical to prevent degradation over a wide voltage window, which can be tackled by carefully selecting chemically stable polymers and additives. Compatibility with electrodes remains another significant hurdle, requiring good interfacial interaction to reduce resistance and prevent dendrite growth; various surface modifications can improve compatibility. Furthermore, addressing environmental and safety concerns is paramount; using non-toxic, biodegradable, and non-flammable materials in GPE formulations can mitigate risks associated with thermal and mechanical stresses. Cost and scalability also pose challenges, necessitating the development of economical production methods that utilize readily available materials and simplify the synthesis process for commercial viability. Lastly, ensuring long-term performance consistency is vital, as GPEs must endure the extended life cycles demanded by practical applications, requiring comprehensive testing and optimization of both polymer compositions and electrolyte formulations. These challenges collectively shape the research efforts to optimize GPEs for reliable use in advanced lithium-ion batteries.

### 6.6. Formulation of GPEs and Effect on Battery Performance

#### 6.6.1. Solution Casting

GPEs are formulated using various techniques, each with unique implications for their performance in lithium-ion batteries. A commonly employed method is solution casting, where the polymer matrix, often PVDF or PEO, is dissolved along with lithium salts and plasticizers in a solvent. This solution is cast into a film and the solvent is evaporated, which results in a homogeneous and flexible electrolyte layer. The solution casting method allows for precise control over the polymer and salt composition, enabling the tailoring of mechanical properties and ionic conductivity to enhance battery performance. Solution-cast gel polymer electrolytes (GPEs) provide several advantages. They are straightforward to produce, flexible, and compact, with the capability to adjust their weight and thickness. These electrolytes are pressure-resistant and non-volatile. However, the low crystallinity and salt presence may lead to reduced mechanical strength in solution-cast GPEs. To improve their mechanical properties, crosslinking agents can be introduced to link the polymer chains within the matrix [107,108].

#### 6.6.2. In Situ Polymerization

Another innovative approach is in situ polymerization, where monomers polymerize directly on the electrodes within the battery cell. This method forms a gel phase that adheres well to electrode surfaces, improving the electrolyte–electrode interface, a critical factor for efficient ion transport and electrochemical stability. In situ polymerized GPEs typically exhibit enhanced mechanical integrity and electrochemical performance, partly due to their uniform structure and intimate contact with electrode materials [109,110]. Coating the premixture onto the electrode followed by polymerization can significantly decrease the contact resistance between the electrode and the electrolyte. This reduction in interfacial resistance helps prevent issues like the formation of lithium dendrites. This method offers several benefits, such as low viscosity, ease of handling, and the creation of an excellent interface [111].

#### 6.6.3. Electrospinning

Electrospinning, a technique that utilizes an electric field to create fine nanofiber mats from polymer solutions, has also been applied to GPEs to increase their surface area and porosity. The resulting nanofibrous structure significantly improves electrolyte uptake and provides a larger surface area for ion conduction, leading to higher ionic conductivities [112,113]. Such enhancements help improve battery efficiency and capacity retention over repeated charge cycles. Each of these formulation methods provides distinct benefits and challenges, affecting crucial performance metrics such as ionic conductivity, mechanical flexibility, thermal stability, and interface interaction in lithium-ion batteries. Therefore, the choice of method is crucial and should align with specific battery performance requirements and application contexts. By innovating new formulations and adapting existing methods, researchers can optimize GPEs to achieve safer, more reliable, and higher-performance lithium-ion batteries.

The characterization of GPEs in LIBs focuses on several key properties to optimize performance. Ionic conductivity is measured using electrochemical impedance spectroscopy to ensure efficient ion transport, while mechanical properties are assessed via tensile testing for strength and flexibility. Thermal stability, crucial for safe operation, is evaluated through thermogravimetric analysis and differential scanning calorimetry. The electrochemical stability window, which determines the safe voltage range, is measured with cyclic voltammetry. To understand the distribution and structure of materials, scanning electron microscopy and X-ray diffraction are employed [114,115]. Interfacial properties with electrodes are assessed using FTIR, Raman spectroscopy, and impedance spectroscopy [116]. Additionally, solvent uptake and swelling behavior are measured by observing weight changes after solvent immersion to ensure the GPE maintains ionic conductivity. These analyses collectively enable the fine-tuning of GPEs for enhanced battery performance.

Conclusively, in reviewing the manuscript, it is important to consider the unique properties of various gel types and their impact on energy storage applications. Polymeric gels, for example, exhibit good mechanical flexibility and ionic conductivity, making them suitable for battery applications that require durability and efficient ion transport. In contrast, silica-based gels provide enhanced thermal stability and a more rigid structure, which can be advantageous in high-temperature environments; however, their brittleness may restrict their use in flexible battery designs. We believe that polymeric gels generally offer more advantages due to their tunable properties, which can be adjusted by selecting different monomers and crosslinking densities. Additionally, their ability to create a cohesive interface with electrode materials enhances performance in terms of energy density and cycle life. Ultimately, the selection of the appropriate gel type should align with the specific requirements of the intended application, carefully balancing factors such as ionic conductivity, mechanical stability, thermal stability, and desired flexibility to achieve more effective energy storage solutions.

## 7. Conclusions and Future Direction

Gel polymer electrolytes (GPEs) present a promising alternative to liquid organic electrolytes for lithium-ion batteries, offering substantial improvements in safety, long-term cycling stability, and adaptability. Unlike liquid electrolytes, which are prone to leakage and flammability, GPEs have a solid-like nature that mitigates these risks, enhancing the battery’s safety profile significantly. Additionally, GPEs exhibit superior cycling stability due to their ability to suppress lithium dendrite formation, a common issue that often leads to short circuits in liquid electrolyte-based batteries. This suppression results in a longer battery lifespan, which is crucial for the reliability of consumer electronics and electric vehicles. Electrochemically, GPEs provide ionic conductivity levels comparable to, or exceeding, those of liquid electrolytes, and maintain performance over a wide temperature range. Their flexible lightweight nature supports innovative battery designs, essential for modern technology applications such as wearable and implantable devices. Nevertheless, challenges remain in optimizing GPE formulations to balance mechanical strength, ionic conductivity, and interfacial stability with electrodes. Integrating high-performance cathode materials such as nickel cobalt aluminum oxide (NCA), nickel cobalt manganese (NCM), and lithium iron phosphate (LiFePO_4_) with GPEs presents an exciting development. NCA and NCM cathodes, known for their high energy density, combined with the safety and stability of GPEs, could significantly enhance battery performance and longevity. LiFePO_4_ cathodes, prized for their thermal stability and safety, could further benefit from the use of GPEs, resulting in batteries with remarkable reliability and safety profiles.

Future research should focus on developing advanced materials and novel composites that enhance the functional properties of GPEs, as along with exploring scalable manufacturing techniques to ensure commercial viability. Continued multidisciplinary research and advancements in polymer chemistry, nanotechnology, and material science are anticipated to further enhance the performance and application range of GPEs. Future research on gel polymer electrolytes (GPEs) should concentrate on their integration into next-generation battery technologies, particularly solid-state and lithium-sulfur batteries. This effort should aim to optimize GPE formulations to enhance both ionic conductivity and mechanical integrity, addressing critical issues such as polysulfide dissolution and interfacial compatibility. Additionally, investigations into the thermal properties of GPEs will be essential to improve safety and efficiency, enabling these batteries to function reliably in diverse conditions. By conducting extensive tests on the cycling performance of GPEs, researchers can pave the way for innovative energy storage solutions that are safer, more efficient, and adaptable to evolving energy needs. Ultimately, this focus on the integration of GPEs could make them crucial components in the advancement of future energy storage technologies.

## Figures and Tables

**Table 1:** Gel polymer electrolytes and application in lithium-ion battery.

Polymer Backbone	Chemical Structure	Key Properties	References
Polyvinylidene Fluoride (PVDF)	-(CH_2_-CF_2_)_n_-	High chemical and thermal stability, good mechanical properties, and electrochemical stability	[15]
Polyacrylonitrile (PAN)	-(CH_2_-CH(CN))_n_-	Good thermal stability, high ionic conductivity with plasticizers and salts	[16]
Polymethyl Methacrylate (PMMA)	-(CH_2_-C(CH_3_)(COOCH_3_))_n_-	Good mechanical strength, transparency, and stable matrix	[17]
Polyvinyl Alcohol (PVA)	-(CH_2_-CHOH)_n_-	Good film-forming properties, highly flexible, can form hydrogels	[18]
Polypropylene Oxide (PPO)	-(CH_2_-CH(CH_3_)-O)_n_-	Good flexibility, high ionic conductivity, good solvating power for salts	[19]

**Table 2:** Durability and lifecycle of high-voltage gel polymer electrolyte at various temperatures.

Polymer Marix	Liquide Electrolyte	Cathode Name	Voltage Range	Cycle Life	Reference
GPE PAN_LAGP_PEGDA	LiTFSI/DMF	NCM-811	2.8–4.3	270 @ 0.5C	[78]
GPE PVDF-HFP_BaTiO_3_	LiPF_6_/EC/DMC	NCM-532	2.5–4.5	200 @ 5C	[79]
GPEMMA_TEGDMA_ETA	LiDFOB/FEC	NCA	3–4.5	200 @ 0.2C	[80]
GPE UPyMA_TEGDA	LiFSI/FEC/EMC/EGA	NCM-811	2.8–4.7	500 @ 0.5C	[81]
GPE LiNO_3__DMAA	LiPF_6_ /EC/DEC/FEC	NCM-622	3–4.3	400 @ 1C	[82]
GPE DOL-PEE	LiTFSI/LiDFOB/FEC	NCM-622	-	300 @ 0.5C	[83]
GPE TFPO-PEE	LiDFOB/LiTFSI	NCM-622	2.7–4.5	300 @ 0.5C	[84]
GPE PEGDA_PETA_PPO	LiPF_6_/EMC/FEC	NCM-811	2.8–4.3	400 @ 1C	[85]
GPE PMBA	LiTFSILiBOB/EC/EMC	NCM-622	3.0–4.3	700 @ 1C	[86]
SPE PVC-TF_3_	LiTFSI/NMP	NCM-811	2.5–4.3	150 @ 0.1C	[87]
SPE B-PEGMA_VC_AN	LiTFSI	NCM-811	2.8–4.3	100 @ 0.5C	[88]
CPE LATP_PEO_PAN	LiTFSI/DMF	NCM-622	2.8–4.3	120 @ 0.5C	[89]

## Data Availability

All data and materials are available on request from the corresponding author.
